# Recently developed radiopharmaceuticals for bacterial infection imaging

**DOI:** 10.1186/s41181-024-00279-7

**Published:** 2024-06-19

**Authors:** Maryke Kahts, Beverley Summers, Aadil Gutta, Wilfrid Pilloy, Thomas Ebenhan

**Affiliations:** 1 Pharmaceutical Sciences Department, School of Pharmacy, Sefako Makgatho Health Sciences University, Ga-Rankuwa, 0208 South Africa; 2 Nuclear Medicine Department, Dr George Mukhari Academic Hospital, Ga-Rankuwa, 0208 South Africa; 3 School of Medicine, Sefako Makgatho Health Sciences University, Ga-Rankuwa, 0208 South Africa; 4 Nuclear Medicine Department and Nuclear Medicine Research Infrastructure, University of Pretoria, Pretoria, 0001 South Africa

**Keywords:** Bacterial infection imaging, Radiolabelled bacterial siderophores, Radiolabelled antimicrobial peptides, Radiolabelled antibiotics, Radiolabelled sugar molecules, Targeting bacterial nitro, Reductase, Targeting bacterial synthesis pathways

## Abstract

**Background:**

Infection remains a major cause of morbidity and mortality, regardless of advances in antimicrobial therapy and improved knowledge of microorganisms. With the major global threat posed by antimicrobial resistance, fast and accurate diagnosis of infections, and the reliable identification of intractable infection, are becoming more crucial for effective treatment and the application of antibiotic stewardship. Molecular imaging with the use of nuclear medicine allows early detection and localisation of infection and inflammatory processes, as well as accurate monitoring of treatment response. There has been a continuous search for more specific radiopharmaceuticals to be utilised for infection imaging. This review summarises the most prominent discoveries in specifically bacterial infection imaging agents over the last five years, since 2019.

**Main body:**

Some promising new radiopharmaceuticals evaluated in patient studies are reported here, including radiolabelled bacterial siderophores like [^68^Ga]Ga-DFO-B, radiolabelled antimicrobial peptide/peptide fragments like [^68^Ga]Ga-NOTA-UBI29-41, and agents that target bacterial synthesis pathways (folic acid and peptidoglycan) like [^11^C]para-aminobenzoic acid and D-methyl-[^11^C]-methionine, with clinical trials underway for [^18^F]fluorodeoxy-sorbitol, as well as for ^11^C- and ^18^F-labelled trimethoprim.

**Conclusion:**

It is evident that a great deal of effort has gone into the development of new radiopharmaceuticals for infection imaging over the last few years, with remarkable progress in preclinical investigations. However, translation to clinical trials, and eventually clinical Nuclear Medicine practice, is apparently slow. It is the authors’ opinion that a more structured and harmonised preclinical setting and well-designed clinical investigations are the key to reliably evaluate the true potential of the newly proposed infection imaging agents.

## Background

Infection remains a global threat and a major cause of morbidity and mortality, regardless of advances in antimicrobial therapy and improved knowledge of microorganisms. Early, accurate diagnosis and localisation of infection can be challenging, invasive and time-consuming, with possible resultant life-threatening delays in appropriate treatment commencement (Akter et al. 2023; Holcman et al. 2019, 2023; Jasińska et al. 2022; Burroni and Evangelista 2021; Albano et al. 2020; Giraudo et al. 2020; Mota et al. 2020a; Palestro 2019, 2020; Meyer et al. 2019). The prediction is that the major cause of death, globally by 2050, will be drug-resistant infections (Akter et al. 2023; Mota et al. 2020a; Kleynhans et al. 2023; Signore et al. 2023; Jain 2022; Fang et al. 2020). With the major threat posed by antimicrobial resistance to the healthcare system and the effective treatment of infections, fast and accurate diagnosis of infections, and the reliable identification of intractable and resistant infection, are becoming more crucial for the application of antibiotic stewardship and to avoid overuse of broad-spectrum antibiotics (Akter et al. 2023; Mota et al. 2020a; Fang et al. 2020; Glaudemans and Gheysens 2023; Gouws et al. 2022; Pijl et al. 2021; Welling et al. 2019).

Imaging is often performed to aid in the localisation of infection or inflammation and to determine the extent of tissue or organ involvement (Jasińska et al. 2022; Giraudo et al. 2020; Mota et al. 2020a; Palestro 2019, 2020; Meyer et al. 2019; Kleynhans et al. 2023). Anatomical radiographic imaging lacks initial sensitivity to detect infection and has no specificity to differentiate between various infectious diseases, as morphologic changes often occur in the later stages of infection (Akter et al. 2023; Jasińska et al. 2022; Giraudo et al. 2020; Mota et al. 2020a; Palestro 2019; Signore et al. 2023; Pijl et al. 2021; Seltzer et al. 2019). However, molecular imaging with the use of nuclear medicine reflects pathophysiological processes and changes, allowing early detection and localisation of infection and inflammatory processes, as well as accurate monitoring of treatment response (Holcman et al. 2023; Jasińska et al. 2022; Albano et al. 2020; Giraudo et al. 2020; Palestro 2019, 2020; Meyer et al. 2019; Kleynhans et al. 2023; Signore et al. 2023; Glaudemans and Gheysens 2023; Pijl et al. 2021; Seltzer et al. 2019). Since the discovery of gallium-67 for medical use in the 1940s (Dittrich and Jesus 2022) and, later on, autologous leukocytes radiolabelled with indium-111, reported for the first time in 1976 (Segal et al. 1976), and technetium-99m, a few years later, for infection imaging, there has been a continuous search for more bacteria-specific radiopharmaceuticals to be utilised for infection imaging (Akter et al. 2023; Palestro 2019; Glaudemans and Gheysens 2023; Gouws et al. 2022; Pijl et al. 2021; Dadachova and Rangel 2022; Signore et al. 2022; Koźmiński et al. 2021). Conventional infection imaging agents (such as [^67^Ga]Ga-citrate, [^111^In]In-oxine- or [^99m^Tc]Tc-HMPAO-labelled leukocytes, and [^18^F]FDG) do not target bacteria, but rather the host immune response to the bacterial infection, and will therefore also localise in sterile inflammatory foci. The nonspecificity of these tracers can result in delayed or even inaccurate diagnosis, localisation, and treatment of infection, which may increase the cost-related burden, antimicrobial resistance, morbidity and even mortality of patients (Jiang et al. 2024; Margeta et al. 2024; Santos et al. 2024; Spoelstra et al. 2024; Li et al. 2020). The development and investigation of new radiopharmaceuticals focuses on the differentiation of infection from sterile inflammation and the imaging of specific infectious microbes and processes (Palestro 2019; Signore et al. 2022). The higher sensitivity and diagnostic value of positron emission tomography (PET) over single photon emission computed tomography (SPECT) has led to an increased focus on new PET radiopharmaceuticals for infection imaging (Kleynhans et al. 2023), but some research is also being conducted on the search for more specific ^99m^Tc-labelled infection imaging agents, due to its wide availability from in-house molybdenum-99/technetium-99m generators and ideal imaging characteristics for SPECT (Akter et al. 2023; Fang et al. 2020; Signore et al. 2022).

Current reviews have discussed the requirements of and provided recommendations to improve the search for infection-specific imaging agents based on the research findings from the last two decades (Mota et al. 2020a; Palestro 2019; Kleynhans et al. 2023; Welling et al. 2019; Dadachova and Rangel 2022; Signore et al. 2022; Polvoy et al. 2020; Sethi et al. 2019). This review rather aims to highlight the most prominent discoveries from the past five years, regarding radiopharmaceutical development and characterization of bacterial infection imaging agents.

## Main text

### Radiolabelled bacterial siderophores

**Fig. 1:**
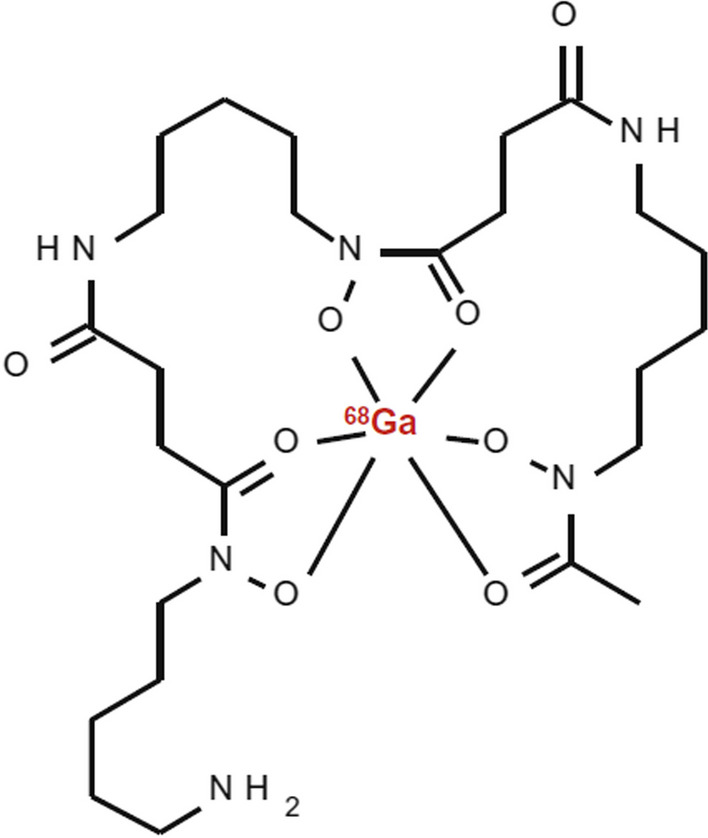
Structure of [^68^Ga]Ga-DFO-B (Petrik et al. 2021)

Bacteria produce and secrete siderophores, which are small molecules with strong iron-binding ability, and express active siderophore uptake receptors on their cell surfaces to obtain and internalise siderophore-chelated iron from human blood (Akter et al. 2023; Kleynhans et al. 2023). Thus, radiolabelled bacteria-specific siderophores may be considered a new class of molecules for prospective imaging of infection. Research by Petrik et al. (2021) suggested that gallium-68, a ferric-like ion analogue, can be utilized for the radiosynthesis of certain bacterial siderophores and thereby exploited the bacterial pathway of the acquisition and storage of iron to visualize bacterial infections using PET/CT imaging. Evidently, desferrioxamine-B (DFO-B) is a hydroxamate siderophore registered for long-term metal chelation therapy in a variety of diseases. Iron is tightly bound to haemoglobin, transferrin, lactoferrin, etc. in the human body. Petrik et al. (2021) synthesized [^68^Ga]Ga-DFO-B (see Fig. 1) and characterized it in vivo using acute murine myositis and respiratory rat models. The authors herein reported sensitive and specific accumulation of [^68^Ga]Ga-DFO-B in *Pseudomonas aeruginosa (P. aeruginosa)* and *Staphylococcus aureus (S. aureus)*, as well as specific uptake in the infection sites of the animals, with rapid renal excretion and minimal localisation in other organs. These are promising preclinical results which motivated for a recently proposed clinical study in patients with vascular graft infections (Akter et al. 2023). For further information, a comprehensive review on radiometal complexation with a particular focus on bacterial and fungal siderophores as cellular targets, was also published by Akter and colleagues (Akter et al. 2023).

**Fig. 2:**
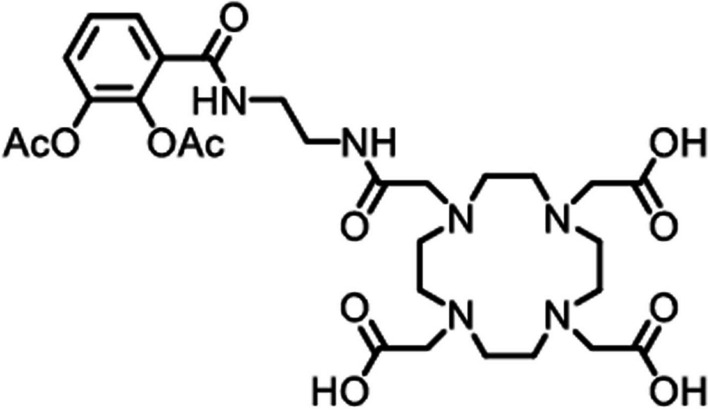
Structure of compound 7 synthesised by Peukert et al. (2021)

Peukert et al. (2021) optimised a number of synthetic siderophores for complexation with ^68^Ga and investigated their bacterial uptake. Compounds-7 (see Fig. 2) and -15 (refer to the original publication for full names) labelled with ^68^Ga illustrated the most promising in vitro results and were investigated in an *Escherichia coli (E. coli)*-induced myositis mouse model. In particular, [^68^Ga]Ga-compound-7 illustrated superior in vivo performance with an 11-fold increased accumulation in the infected muscle when compared to healthy muscle tissue. Rapid uptake of [^68^Ga]Ga-compound-7 in both the infected and (sterile) inflamed muscle tissue was observed, with significant washout from the sterile inflammatory focus over time.

**Fig. 3:**
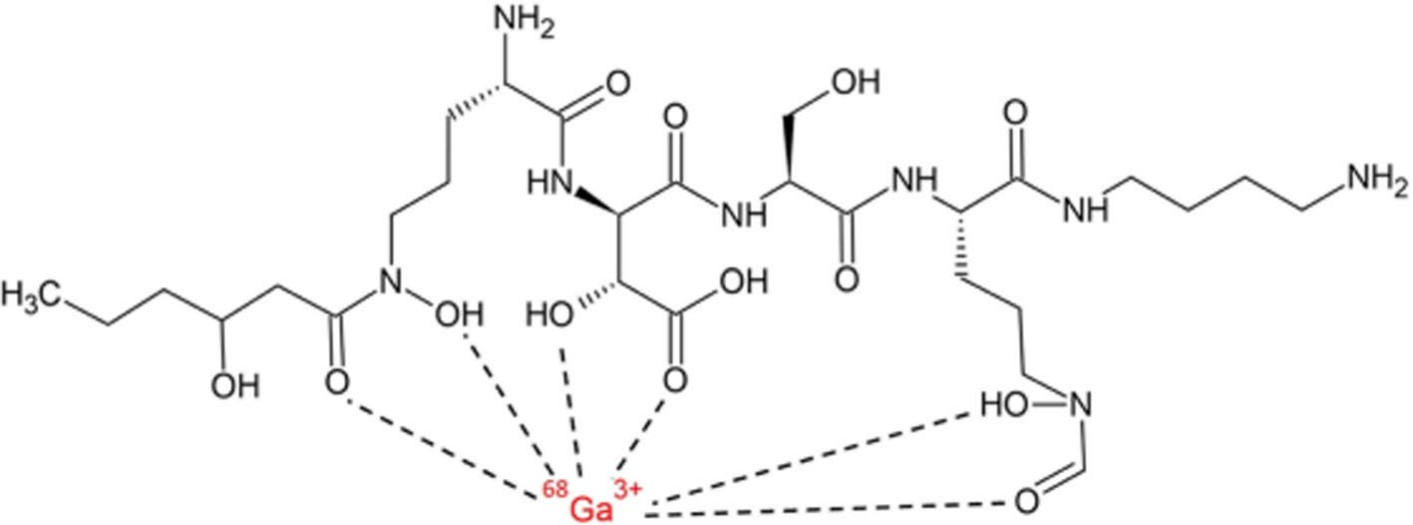
Structure of [^68^Ga]Ga-ORNB-C6 (Bendova et al. 2023)

Bendova et al. (2023) radiolabelled a variant of ornibactin (ORNB-C6) with ^68^Ga to synthesise [^68^Ga]Ga-ORNB-C6 (see Fig. 3) with a high radiochemical purity (> 95%) and high in vitro stability in human serum over 2 h (> 97%). Ornibactin is a siderophore produced by *Burkholderia cepacia* complex (BCC) bacteria, which are pathogens involved in the development of hospital-acquired pneumonia in immunocompromised patients.

In vitro uptake studies of [^68^Ga]Ga-ORNB-C6 in different BCC cultures illustrated significantly high uptake in *B. multivorans* species and comparative uptake studies with other respiratory pathogens showed significantly lower uptake in only *S. aureus* and *P. aeruginosa* cultures. Animal biodistribution studies in a *B. multivorans*-induced myositis mouse model illustrated rapid renal excretion of the tracer and significantly increased accumulation of [^68^Ga]Ga-ORNB-C6 in the infected muscle tissue when compared to healthy muscle tissue, which was further significantly increased in immunosuppressed mice (n = 3 per group). These biodistribution results were confirmed with animal PET/CT imaging performed 45 min after tracer injection. The authors reported sensitive and specific accumulation of [^68^Ga]Ga-ORNB-C6 in the *B. multivorans* infectious foci (which was further confirmed in PET/CT imaging of a rat model of pulmonary infection), with no accumulation in sterile inflammatory foci, heat-inactivated *B. multivorans* or *E. coli*-infected sites.

**Fig. 4:**
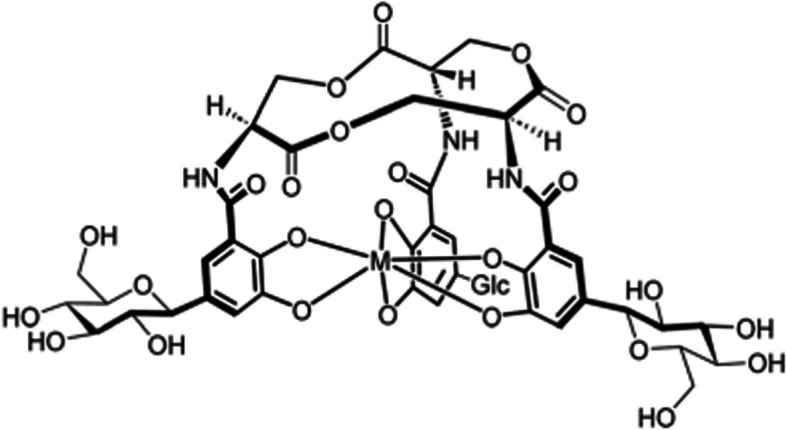
Structure of [^68^Ga]Ga-RMA693 (M = ^68^Ga) (Margeta et al. 2024)

Margeta et al. (Margeta et al. 2024) utilised salmochelin, an enterobactin-based siderophore, to radiolabel the salmochelin-derivate, RMA693, with ^68^Ga with the aim of targeting *E. coli*-induced infections. The synthesis of the precursor seems quite complicated, but subsequent [^68^Ga]Ga-RMA693 (see Fig. 4) radiosynthesis was performed within 10 min and yielded radiochemical purities > 99%.

In vitro bacterial uptake studies demonstrated specificity for bacteria that utilises the salmochelin-based transporter system, i.e. selected pathogenic *E. coli* and *S. aureus* strains. In vivo [^68^Ga]Ga-RMA693-PET/CT biodistribution studies in healthy adult mice illustrated rapid renal excretion of the tracer (> 90% of the injected dose at 90 min post-injection) with no accumulation in other organs or tissues. PET/CT imaging was also performed in a mouse model infected with *E. coli* ATCC25922, and *E. coli* TG1 as a negative control, which demonstrated specific, bacterial-load correlated and strain-dependent accumulation of [^68^Ga]Ga-RMA693 in the *E. coli* ATCC25922-infected muscle at 90 min post-injection of the tracer.

**Fig. 5:**
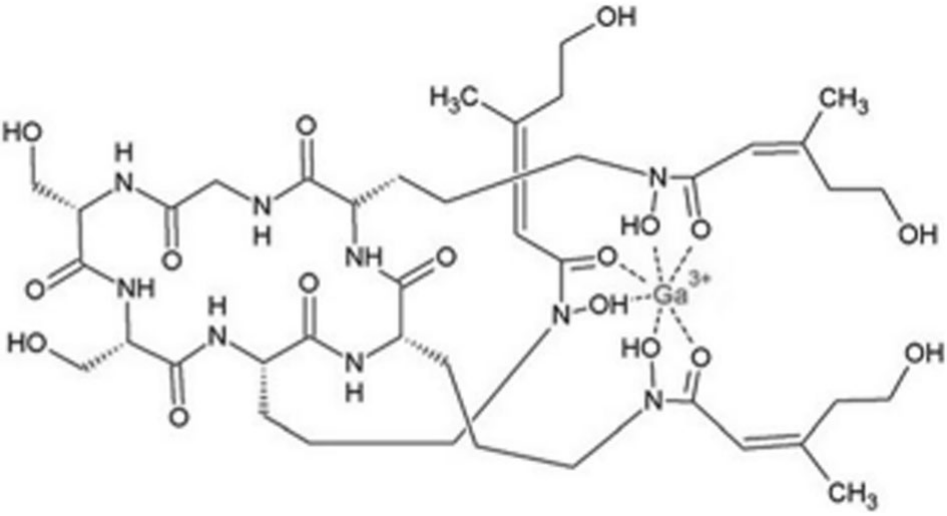
Structure of [^68^Ga]Ga-FR and [^68^Ga]Ga-FRH (Krasulova et al. 2024)

Most recently, Krasulova et al. (2024) performed the radiolabelling of two fungal siderophores, i.e. ferrirhodin (FRH) and ferrirubin (FR), with ^68^Ga, investigated their in vitro uptake in bacterial cell cultures and ex vivo biodistribution in a healthy animal model, and performed small animal PET imaging in a mouse model of infection. The radiosynthesis of [^68^Ga]Ga-FRH and [^68^Ga]Ga-FR (see Fig. 5) yielded high radiochemical purities (> 95%) and both tracers demonstrated high stability in human serum (99% for [^68^Ga]Ga-FRH and 90% for [^68^Ga]Ga-FR after a 2-h incubation period).

[^68^Ga]Ga-FRH illustrated higher plasma protein binding (approximately 50%) than [^68^Ga]Ga-FR (6%) after a 2-h incubation period. In vitro bacterial uptake studies illustrated uptake of both tracers in *S. aureus*, *P. aeruginosa* and *K. pneumoniae* with negligible uptake observed in *E. coli* and *Candida albicans* (fungi) cultures. The biodistribution of [^68^Ga]Ga-FR in a healthy mouse model yielded more promising results than [^68^Ga]Ga-FRH, with rapid renal clearance, low accumulation and rapid clearance from other organs, and low blood pool retention. [^68^Ga]Ga-FRH illustrated a higher blood pool retention and increased accumulation in perfused organs, with the highest radioactivity observed in the kidneys and lungs. These biodistribution results were confirmed with small animal PET/CT imaging in a healthy mouse model. [^68^Ga]Ga-FR outperformed [^68^Ga]Ga-FRH as a potentially promising PET infection imaging agent as illustrated in a *S. aureus*-induced myositis mouse model, with significant signal accumulation in the infectious foci and no accumulation in heat-inactivated bacteria. [^68^Ga]Ga-FRH also showed tracer accumulation in the infectious foci, but was indistinguishable from the PET signals in other organs.

**Fig. 6:**
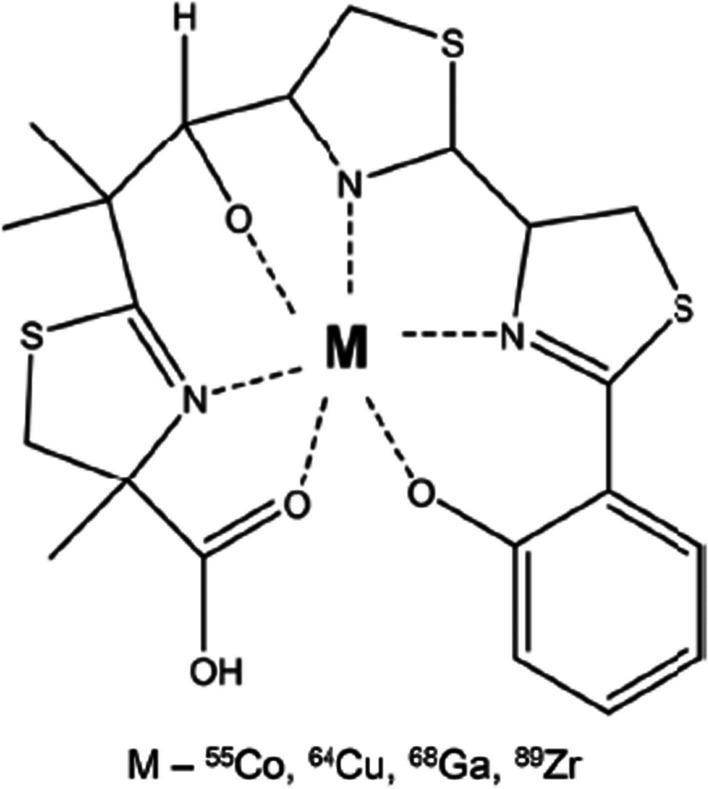
Structure of metal-YbT complex (Siddiqui et al. 2021)

Ongoing research is investigating the potential of other metal radionuclides, like ^64^Cu and ^89^Zr, for the siderophore shuttle pathway (Kleynhans et al. 2023). Siddiqui et al. (2021) radiolabelled the copper-binding siderophore, yersiniabactin (YbT) (found in *E. coli* and *Klebsiella pneumoniae*), with ^64^Cu, ^57^Co, ^89^Zr and ^68^Ga, respectively (see Fig. 6).

Conveniently, the authors developed a one-step radiolabelling process, where 10 μg YbT in 50 μL buffered media was added to 50 μCi of the radiometal and the radiolabelled products were achieved after incubation at 37 °C for 1 h or at 90 °C for 30 min. The authors observed superior complexation of YbT with ^64^Cu and further investigated the in vitro stability of [^64^Cu]Cu-YbT in mouse serum (60% at 4 h), and assured compound specificity for *E. coli* by using a myositis mouse model ([^64^Cu]Cu-YbT showed no uptake in the negative controls, i.e. *S. aureus* and *P. aeruginosa*). The tracer was also able to detect small bacterial burden (> 10^4^ cfu) and demonstrated accurate disease representation on PET images that correlated with decreased bacterial counts after administration of ciprofloxacin. The results presented by Siddiqui et al. (2021) warrant potential for [^64^Cu]Cu-YbT-PET/CT in becoming a novel tool for accurate, sensitive and bacteria-specific imaging, as well as an imaging biomarker regarding monitoring certain antibiotic treatment efficacy.

### Radiolabelled antimicrobial peptides/peptide fragments

Antimicrobial peptides (AMPs) are small, positively charged biopeptides that form part of the innate immune system in living organisms. They exhibit broad-spectrum antimicrobial activity, generally through their electrostatic adsorption to the negatively charged domains of bacterial cell membranes. The advantages of antimicrobial peptides include their negligible localisation in host cells, which translates to bacteria-specific uptake and a low risk of cytotoxicity, as well as their lower immunogenicity when compared to proteins. AMPs are therefore highly desired vectors qualifying for infection-selective imaging which necessitates their development as PET/SPECT radiopharmaceuticals. Some potential disadvantages of antimicrobial peptides include their short half-life and decreased antibacterial activity in vivo due to proteolytic enzyme degradation and their susceptibility to pH changes in the microenvironment (Xuan et al. 2023). Recent developments in the past 5 years mainly featured ^68^ Ga-, ^99m^Tc-, and ^64^Cu-labelled AMPs.

### ^***68***^***Ga-labelled antimicrobial peptide fragments***

Ubiquicidin 29–41 (UBI29-41) is one of the smaller, synthetic, cationic AMP fragments of the UBI(1–59) protein, only consisting of 12 amino acids with documented specificity for bacterial cells, and negligible binding to mammalian or cancer cells (Boddeti and Kumar 2021). This peptide fragment has therefore been investigated quite extensively for the imaging of bacterial infections. More recently, clinical investigations have focussed on developing a ^68^Ga-radiosynthesis of UBI29-41, as well as UBI31-38, by way of using bifunctional chelators, for example 1,4,7-triazacylonane- 1,4,7-triacetic acid (NOTA), ultimately allowing the utilization of PET instead of SPECT for more sensitive infection imaging (Kleynhans et al. 2023; Marjanovic-Painter et al. 2023).

Perhaps the most recognized clinical investigation was mentioned in a meeting report by Vilche et al. (2019), comparing the diagnostic value of [^68^Ga]Ga-NOTA-UBI29-41-PET against [^18^F]FDG-PET for differentiation between infection and aseptic hip implant loosening in 21 patients. The authors reported higher sensitivity (93%) and specificity (100%) for [^68^Ga]Ga-NOTA-UBI29-41-PET, compared to [^18^F]FDG-PET in this patient population. The abstract also mentions the first GMP-compliant [^68^Ga]Ga-NOTA-UBI29-41 production using a synthesis module for ^68^Ga-radiolabelling (Roux et al. 2020). The authors mentioned that more patients will be investigated, but no new results have been published to date.

Santos et al. (2024) recently developed a novel method for in-house production of [^68^Ga]Ga-NOTA-UBI29-41, utilising a titanium dioxide column-based generator. The generator was eluted with 0.1 M HCl and the eluate pushed through a SCX resin to remove Zn^2+^, Ti^2+^ and ^68^Ge impurities and to concentrate the eluate, followed by the elution of the cationic resin filter with pure acetone to obtain ^68^Ga for [^68^Ga]Ga-NOTA-UBI29-41 radiosynthesis utilising a NOTA-UBI29-41 in sodium acetate solution. After radiosynthesis, the [^68^Ga]Ga-NOTA-UBI29-41 was purified with a preconditioned (5 mL 50% EtOH/H_2_O and then 5 mL water) Sep-Pak C18 cartridge, recovered with a 70% EtOH/H_2_O solution and diluted with saline. The authors optimised the radiosynthesis parameters for optimal radiochemical yield and purity at a pH of 3.5–4.0, incubation at 85 °C for 5 min and a precursor amount of 20 μg. [^68^Ga]Ga-NOTA-UBI29-41 demonstrated high radiochemical stability (> 98%) at room temperature and under refrigeration over 90 min. However, a reduction in radiochemical stability from 95% at 30 min to 80% at 90 min in human serum was reported, which motivates for the completion of [^68^Ga]Ga-NOTA-UBI29-41-PET studies within 1 h post-injection. The authors reported a 51–61% range of serum protein binding of the tracer. There was high, bacterial load-dependent binding affinity of [^68^Ga]Ga-NOTA-UBI29-41 to *S. aureus* bacterial cells, with the highest affinity (approximately 90%) reported at a bacterial concentration of 6 × 10^9^ CFU/mL.

Boddeti and Kumar (2021) utilised 1,4,7,10-tetraazacycododecane-1,4,7,10-tetraacetic acid (DOTA) as bifunctional chelator to perform ^68^Ga-radiolabelling of UBI29-41 within 15 min, also employing an automated synthesis method, reporting radiochemical purities > 99%, which remained high at 98% for up to 3 h after synthesis. This led the authors to negate the ^68^Ga-product purification step. A radiolabelling yield of > 99% was achieved and product stability in human serum at 37 °C was > 99% after 1 h incubation. The normal biodistribution of [^68^Ga]Ga-DOTA-UBI29-41 in healthy rats illustrated high accumulation in the kidneys and bladder, low accumulation in other tissues and very low background activity after 30 to 60 min. Approximately 50% accumulation of the radiopharmaceutical in *S. aureus* cultures was observed, which remained at 48% for up to two hours after start of incubation. Intense accumulation of [^68^Ga]Ga-DOTA-UBI29-41 was reported in *S. aureus*-infected tissue foci in a rat model at 60 min post-injection. Significant uptake was observed in hepatic tissue early after injection, which washed out over the next 30 min. There was initial accumulation of the tracer in sterile inflammatory tissue foci, which also washed out rapidly.

In 2023, Marjanovic-Painter et al. (2023) provided a critical systematic review concerning the development of available radiolabelled UBI peptide fragments over the last decade. The main conclusion states that these radiopharmaceuticals illustrate promising results as selective, sensitive and infection-specific imaging agents. However, larger clinical studies are needed for their translation to routine Nuclear Medicine practice (Kleynhans et al. 2023).

Chopra et al. (2019) synthesised GF-17, an AMP fragment corresponding to residues 17 to 32 of human antibacterial cathelicidin (LL-37), that was functionalised with [^68^Ga]Ga-DOTA. The radiolabelling parameters were optimised (pH of 4, DOTA-GF-17 = 20 nmol, 95 °C for 30 min) to deliver a labelling efficiency of ≥ 95%. The [^68^Ga]Ga-DOTA-GF-17 complex illustrated good in vitro stability over 3 h and sufficient accumulation in *S. aureus* (69%) and *P. aeruginosa* (44%). Animal PET imaging studies illustrated appropriate uptake in *S. aureus*-infected tissue at 45 min, as well as in *P. aeruginosa*-infected tissue at 120 min.

### ^***99m***^***Tc-labelled antimicrobial peptides***

Human β-defensin 3 (HBD-3) is an AMP with antibacterial activity against both Gram-negative and Gram-positive bacteria. Follacchio et al. (2019) achieved promising results with [^99m^Tc]Tc-HBD-3 in a rat *S. aureus* infection model, with a 5.7-fold higher accumulation in the infectious foci when compared to its uptake in inflammatory tissue. The radiosynthesis hereby provided an acceptable radiolabelling yield of 70%.

**Fig. 7:**
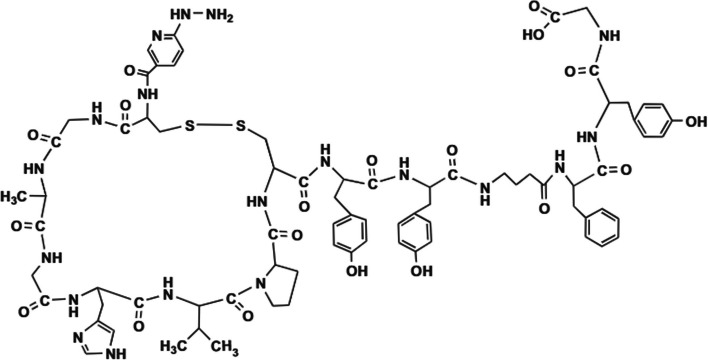
Structure of final peptide synthesised by Tehrani et al. (2021)

MicrocinJ25 (MccJ25) is an AMP produced by some *E. coli* strains with potent inhibitory activities against *E. coli*, *Salmonella* and *Shigella*. Tehrani et al. (2021) utilised a 6-hydrazinonicotinic acid (HYNIC) conjugated cyclic peptide derivative, based on the primary structure of MccJ25 and radiolabelled it with ^99m^Tc using tricine and N,N′-ethylenediamine-diacetic acid (EDDA) as ligands (see Fig. 7).

The [^99m^Tc]Tc-HYNIC/EDDA-MccJ25 conjugate was synthesised with ˃90% radiochemical purity and good stability over 24 h. In vitro accumulation in *E. coli* cultures was 40.5 ± 5.21% following 1 h incubation. Significant tracer accumulation was also observed in a mouse myositis model at 1 h post-injection, along with high renal uptake (route of excretion). [^99m^Tc]Tc-HYNIC/EDDA-MccJ25 SPECT therefore has potential for the specific detection of *E. coli* infections.

**Fig. 8:**
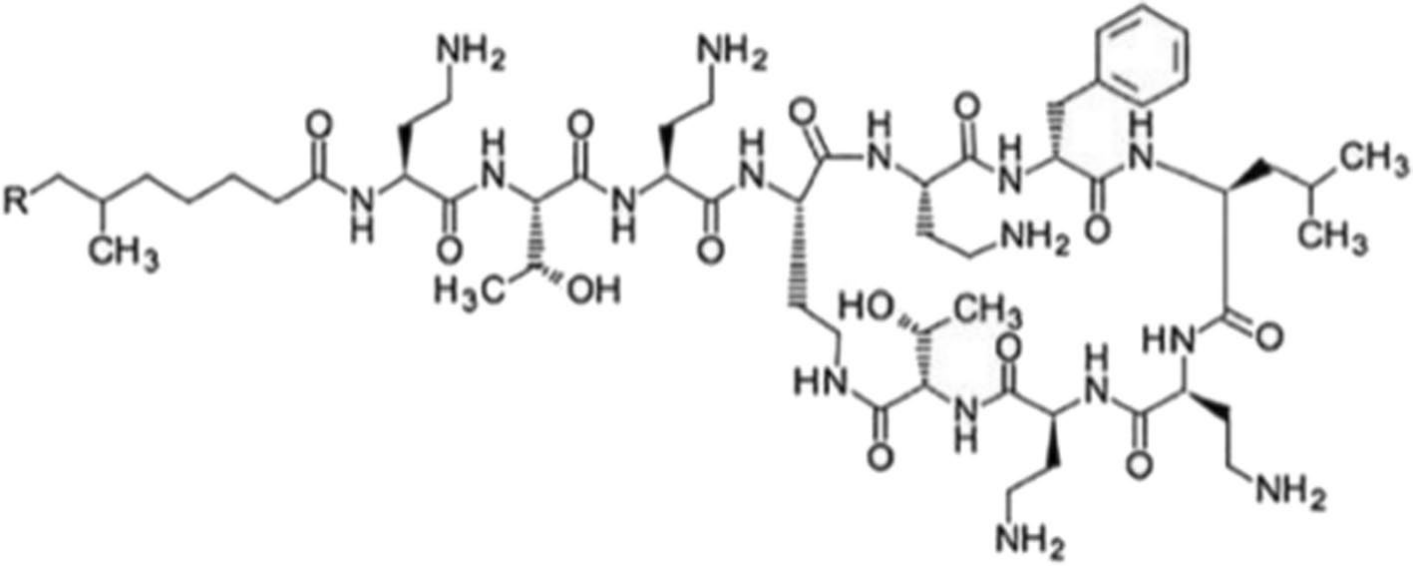
Structure of PMB (Auletta et al. 2021)

Polymyxin B (PMB) is an amphipathic antimicrobial peptide (see Fig. 8) that disrupts the bacterial cell membrane by binding to phospholipids, causing leakage of the bacterial membrane as well as increased vulnerability to hydrophobic antibiotics.

Even though PMB is classified as an antimicrobial peptide, it is commercially available as polymyxin B sulphate and clinically used as an antibiotic in multi-drug resistant Gram-negative bacterial infections, e.g. *P. aeruginosa and A. baumanii* infections. Auletta et al. (2021) radiolabelled polymyxin B sulphate with ^99m^Tc using succinimidyl-6-hydrazinonicotinate hydrochloride (HYNIC) as a bifunctional chelating agent. [^99m^Tc]Tc-HYNIC-PMB was obtained at a labelling efficiency of approximately 97% using a HYNIC:PMB molar ratio of 1.5:1 and demonstrated stability in human serum and saline over 6 h. In vitro bacterial uptake studies demonstrated specific [^99m^Tc]Tc-HYNIC-PMB uptake in Gram-negative bacterial cell cultures (*E. coli*, *P. aeruginosa* and *A. baumanii*) and generally lower uptake in Gram-positive bacterial cell cultures (*K. pneumoniae*, *S. aureus* and *E. faecalis*). In vivo biodistribution studies in a healthy mouse model illustrated highest [^99m^Tc]Tc-HYNIC-PMB accumulation in the liver and kidneys with increasing activity detected in the bladder over time. Bacteria-specific targeting was investigated in a myositis mouse model, which demonstrated significantly increased SPECT signal detection in the *P. aeruginosa*-induced myositis lesion than in *S. aureus*-induced myositis or healthy muscle tissue.

**Fig. 9:**
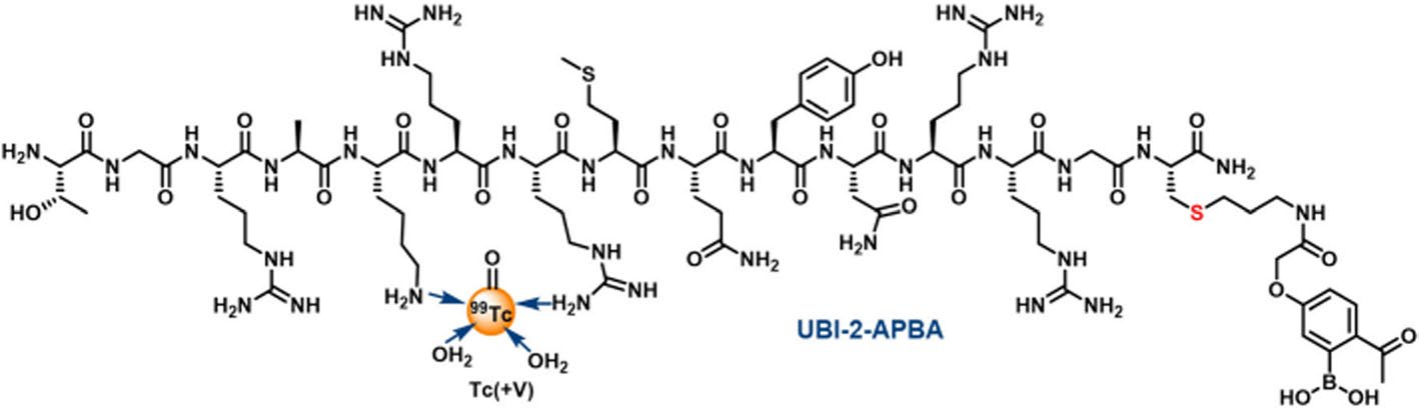
Structure of [^99m^Tc]Tc-UBI29-41–2-APBA (Mitra et al. 2022)

Some promising recent advances have been made with the optimisation of UBI29-41 radiolabelled with ^99m^Tc to improve the tracer’s bacterial binding affinity, where tracer accumulation is not only dependent on the electrostatic interaction with the bacterial cell membrane. Mitra et al. (2022) modified the UBI29-41 peptide with 2-acetyl phenylboronic acid (2-APBA) to allow a synergistic interaction with the primary amine groups expressed on bacterial phospholipids (see Fig. 9).

The authors reported a high radiochemical purity (> 96%) of [^99m^Tc]Tc-UBI29-41-2-APBA and slightly increased stability in human serum and PBS over 16 h when compared to [^99m^Tc]Tc-UBI29-41. [^99m^Tc]Tc-UBI29-41-2-APBA demonstrated a significantly higher in vitro uptake in *S. aureus* cell cultures compared to [^99m^Tc]Tc-UBI29-41, which was hypothesised to be the result of a combination of the stronger covalent binding between 2-APBA and bacterial phospholipids and the weaker electrostatic interaction between positively charged amino acids and bacterial phospholipids. These bacterial uptake results were confirmed with in vivo SPECT imaging in an infection mouse model, where [^99m^Tc]Tc-UBI29-41-2-APBA illustrated improved retention in infectious foci and faster renal clearance of the tracer in comparison with [^99m^Tc]Tc-UBI29-41. [^99m^Tc]Tc-UBI29-41-2-APBA was also able to differentiate between infection and sterile inflammation.

**Fig. 10:**
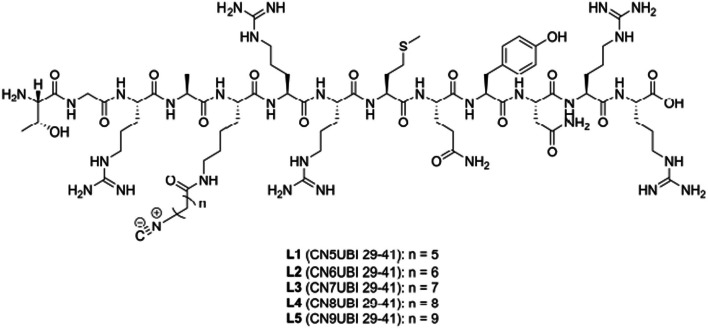
Structures of UBI29-41 isocyanides (Jiang et al. 2024)

Most recently, Jiang et al. (2024) also aimed to improve the accumulation of ^99m^Tc-labelled UBI29-41 in infectious foci and, furthermore, reduce the liver uptake of the tracer by radiolabelling isocyanide UBI29-41 derivatives (CNnUBI29-41; n = 5–9; see Fig. 10) with different ^99m^Tc cores, i.e. [^99m^Tc]Tc(I)^+^, [^99m^Tc][Tc(I)(CO)_3_(H_2_O)_3_]^+^ and [^99m^Tc][Tc(V)N]^2+^. Radiosynthesis of [^99m^Tc]Tc-CNnUBI29-41 complexes were simple and straightforward via a one-step, one-pot reaction utilising kit-based stannous chloride. [^99m^Tc]Tc(CO)_3_-CNnUBI29-41 and [^99m^Tc]TcN-CNnUBI29-41 complexes were radiosynthesised in two-step reactions to allow formation of the ^99m^Tc core and ligand exchange reactions. All tracers were synthesised with radiochemical purities > 90% and demonstrated specific in vitro binding to *S. aureus* cell cultures. Among all the tracers investigated, [^99m^Tc]Tc-CN5UBI29-41 illustrated the highest abscess-to-muscle ratio in biodistribution studies performed in an infection mouse model, which warranted further investigation of this tracer in a mouse infection vs mouse inflammation model. Abscess accumulation of [^99m^Tc]Tc-CN5UBI29-41 in *S. aureus*-infected mice was significantly higher than uptake in turpentine-induced inflammatory foci at 2 h post-injection, which correlated with SPECT imaging results. [^99m^Tc]Tc-CN5UBI29-41 illustrated high blood retention and also showed accumulation in the kidneys, liver, intestines, gallbladder and cardiac blood pool on SPECT images. The authors concluded that the biodistribution and SPECT imaging results still require further investigation and improvement.

### ^***64***^***Cu-labelled antimicrobial peptides***

**Fig. 11:**
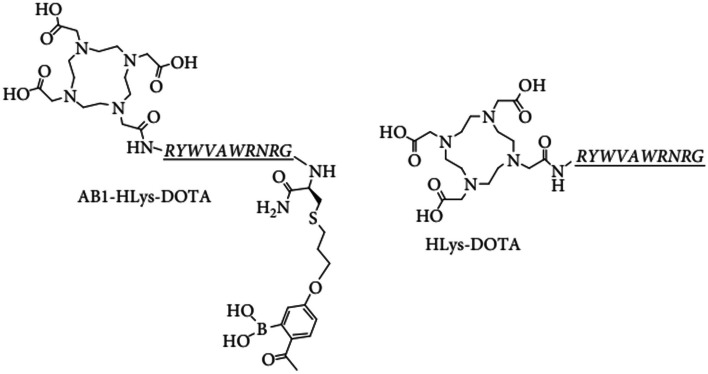
Structures of AB1-HLys-DOTA (left) and HLys-DOTA (right) (Aweda et al. 2019)

Aweda et al. (2019) developed two synthetic short cationic AMPs that bind to amine groups exposed by phosphatidylethanolamine (PE) and lysylphosphatidylglycerol (Lys-PG) of the Gram-positive bacterial cell wall envelope, i.e. D-HLys-DOTA (polycationic decapeptide synthesised as the D-isomer [RYWVAWRNRG] conjugated to DOTA) and AB1-HLys-DOTA (AB1 is an unnatural amino acid that forms covalent bonds with amine groups on PE and Lys-PG through the formation of iminoboronates) (see Fig. 11).

The authors reported on the radiosyntheses of HLys-DOTA and AB1-HLys-DOTA with ^64^Cu and performed PET imaging in an animal model of *S. aureus*-induced murine myositis. The two radiopharmaceuticals were produced with high radiochemical and labelling yields (≥ 95%), though [^64^Cu]Cu-DOTA-HLys-AB1 required C18 SepPak purification. [^64^Cu]Cu-DOTA-HLys-AB1 demonstrated > 98% product stability in PBS, human serum and lysogeny broth, and significantly higher accumulation in *S. aureus* bacterial cells than [^64^Cu]Cu-DOTA-HLys (98.5 ± 3.5% vs. 39.1 ± 3.3%), over 24 h. The specificity of the two radiopharmaceuticals for Gram-positive bacteria was confirmed through a comparison of their accumulation in *P. aeruginosa* (a Gram-negative bacteria), which was 5 to 10 times lower than their uptake in *S. aureus*. The bacteria-specific uptake of [^64^Cu]Cu-DOTA-HLys-AB1 was also confirmed by the determination of mammalian SKBR3 breast cancer cell accumulation of the radiopharmaceutical—9.4 ± 2.3% at 24 h post incubation. Small animal PET/CT imaging revealed significantly higher accumulation of [^64^Cu]Cu-DOTA-HLys-AB1 when compared to [^64^Cu]Cu-DOTA-HLys at 1, 4 and 24 h post administration, with similar mean SUVs between the background activity of [^64^Cu]Cu-DOTA-HLys-AB1 and the accumulation of [^64^Cu]Cu-DOTA-HLys in the infected muscle. Increased accumulation in the liver and kidneys were observed, which points to the fast renal and hepatobiliary clearance of cationic peptides. The authors recommended further studies to evaluate the ability of [^64^Cu]Cu-DOTA-HLys-AB1 to distinguish between infection and sterile inflammation, as well as optimisation of its functional groups for appropriate clearance and increased accumulation in infection foci.

### Bacteria-unique sugars

Clinical microbiology makes use of selective growth media containing small molecules, such as sugars or sugar alcohols, to differentiate between microbes. Researchers have been exploring this known fact in the development of bacteria-specific radiopharmaceuticals, made up of small molecules that are metabolised by bacteria-specific pathways and not mammalian cells (Kleynhans et al. 2023; Jain 2022; Gordon et al. 2019). In particular, maltodextrin transport-based radiopharmaceuticals were pointed out as compounds featuring high in vivo stability, possible imaging of infectious biofilm manifestations, selectivity for bacteria, and their hydrophilic, neutral nature, resulting in fast background clearance (Kleynhans et al. 2023).

### ***[***^***18***^***F]fluorodeoxy-sorbitol (FDS)***

**Fig. 12:**
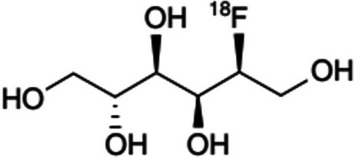
Structure of [^18^F]FDS (Mota et al. 2021)

[^18^F]FDS (see Fig. 12) is localized in bacteria via a metabolically conserved sorbitol-specific pathway. Whilst its first-in-human application was already accomplished in 2008, more recently [^18^F]FDS-PET was employed by Werner et al. (2019) to investigate its potential to offer a more thorough evaluation of human renal kinetics. Furthermore, a well-structured exploratory clinical investigation was published by Ordonez et al. (2021) featuring whole-body [^18^F]FDS-PET/CT in 26 prospectively enrolled patients with confirmed Enterobacterales infection. Enterobacterales is a common pathogen affecting humans and these Gram-negative infections can be life-threatening. Enterobacterales also often causes multi-drug resistant infections. Infection imaging in these patients proved to be accurate and sensitive. The authors also approved tracer selectivity by illustrating increased [^18^F]FDS-PET signals at infection sites whereas minimal signal was noted at the sites of oncologic or sterile inflammatory pathologies. The authors also utilised longitudinal imaging in the same patients to clarify antibiotic efficacy with decreases in PET signal correlating with clinical improvement. These very promising results lead the group to develop a one-step kit for the preparation of [^18^F]FDS within 10 min, utilising commercially available [^18^F]FDG as precursor, which was reduced to [^18^F]FDS at room temperature by macroporous triethylammonium methylpolystyrene borohydride (Mota et al. 2021). The ease of producing [^18^F]FDS from [^18^F]FDG may assist in the future GMP radiosynthesis of [^18^F]FDS.

An [^18^F]FDS-PET clinical trial is currently underway for implementation in Nuclear Medicine clinical practice (Kleynhans et al. 2023). This phase 1 clinical study (NCT05611892) aims to investigate the biodistribution of [^18^F]FDS and pathophysiology determined by [^18^F]FDS-PET/CT imaging in 10 patients with confirmed Enterobacterales infections, invasive fungal infections, oncologic disease or inflammatory disease. The study is currently in the recruitment phase and will be conducted at the Johns Hopkins Medical Institutions, Baltimore, MD, USA.

### ***2-deoxy-[***^***18***^***F]fluoromaltose (FDM) and 2-deoxy-[***^***18***^***F]fluorosakebiose (FSK)***

**Fig. 13:**
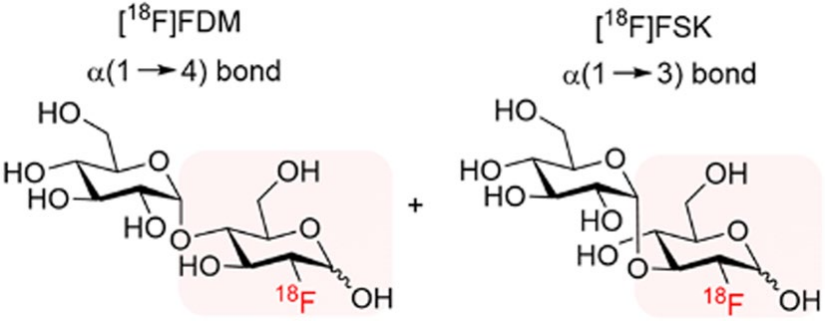
Structures of [^18^F]FDM (right) and [.^18^F]FSK (left) (Sorlin et al. 2023)

The ease of synthesising [^18^F]FDS from commercially available [^18^F]FDG for the specific imaging of Gram-negative bacteria, e.g. *E. coli*-induced infections, lead to Sorlin et al. (2023) exploring a one-step chemoenzymatic (phosphorylase-catalysed) synthesis approach to construct ^18^F-labelled disaccharides from [^18^F]FDG for the potential PET imaging of Gram-positive bacterial infections. The authors initially synthesised [^18^F]FDM (see Fig. 13) from [^18^F]FDG with the aim of targeting the bacterial maltose receptor. The β-D-glucose-1-phosphate precursor was synthesised and a mixture with maltose phosphorylase in citrate buffer prepared. Commercially produced [^18^F]FDG was then directly added to this mixture, followed by stirring for 20 min at 37 °C. [^18^F]FDM was produced with a radiochemical yield of 72%. A by-product of the reaction was [^18^F]FSK (see Fig. 13) with a 15% radiochemical yield (n = 25). In vitro bacterial uptake assays showed high accumulation of both [^18^F]FDM and [^18^F]FSK in *S. aureus* and *K. pneumoniae* cell cultures (uptake similar to [^18^F]FDG), and low uptake in *E. coli*. [^18^F]FDM and [^18^F]FSK demonstrated high stability in human serum, but hydrolysis to [^18^F]FDG in mouse serum, most likely due to increased levels of α-glucosidase, which was demonstrated to be counteracted by the concurrent administration of voglibose. Next, the authors assessed the in vivo accumulation of both tracers in a MRSA-induced myositis mouse model and report significantly increased accumulation of both [^18^F]FDM and [^18^F]FSK in the infected site in comparison with accumulation in heat-killed MRSA, with a slightly better in vivo performance by [^18^F]FSK. [^18^F]FSK was therefore further investigated in an *S. aureus*-induced vertebral discitis-osteomyelitis rat model, as well as an *A. baumannii*-induced myositis mouse model, and demonstrated significantly increased accumulation at the site of infection when compared to the heat-killed bacteria injection site in both cases.

### ***[***^***18***^***F]fluoromaltohexaose (FMH)***

**Fig. 14:**
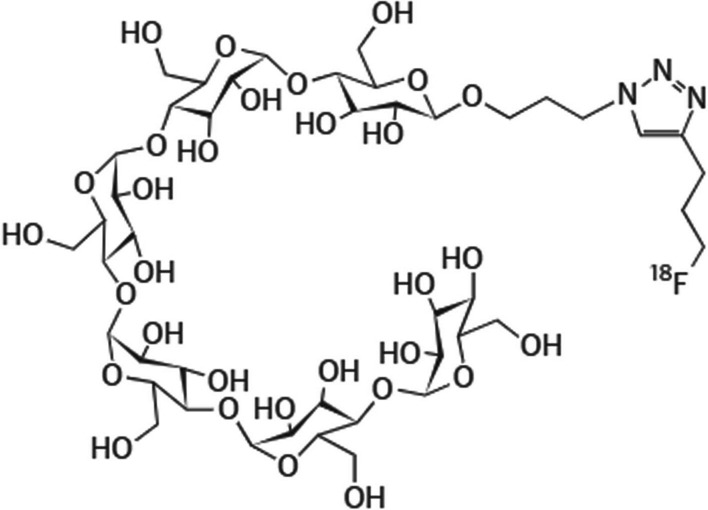
Structure of [^18^F]FMH (Takemiya et al. 2019)

Takemiya et al. (2019) investigated [^18^F]FMH (see Fig. 14) as a potential bacteria-specific PET imaging agent, using a rat model with subclinical *S. aureus*-induced infection of implantable cardiac devices. The [^18^F]FMH radiosynthesis featured a straightforward, one-step fluorination process of brosylate-maltohexaose. The uptake of [^18^F]FMH was compared with [^18^F]FDG. The authors reported significant accumulation of [^18^F]FMH in infected foci when compared with foci of sterile inflammation and unspecific distribution in healthy tissues, whereas there was no significant difference between [^18^F]FDG accumulation in the infected foci and areas of sterile inflammation. The authors concluded that their results illustrate the potential of [^18^F]FMH to detect early-stage infection of implantable cardiac devices, which may allow these infections to be treated with appropriate antibiotics, as well as treatment follow-up monitoring, at an early enough stage so that the device does not have to be removed. No clinical data is available to date.

### ***[***^***18***^***F]fluoromaltotriose***

**Fig. 15:**
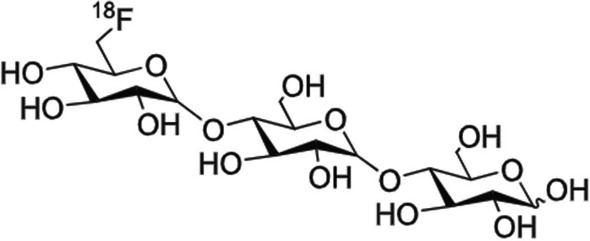
Structure of [^18^F]fluoromaltotriose (Gabr et al. 2020)

Gabr et al. (2020) developed a shortened, more reliable radiosynthesis method for [^18^F]fluoromaltotriose, based on their previous work, with the unambiguous assignment of the ^18^F-position (see Fig. 15). The newly synthesised [^18^F]fluoromaltotriose illustrated 96% stability in human serum over 2 h. In vitro tracer accumulation was performed in *E. coli* cultures, demonstrating 47% cell retention at 30 min. The authors also investigated the specificity of [^18^F]fluoromaltotriose for bacterial infection in a myositis mouse model and reported significantly higher radiopharmaceutical concentration in the infected thigh muscle *versus* the healthy contralateral muscle. A review article by Akter et al. (2023) highlighted the potential of maltotriose as a bacteria-specific ligand, but warned researchers to consider the increased starch-degrading enzyme activity in small animals when compared to human blood, as well as the fact that maltotriose-based radiopharmaceuticals may only localise in metabolically-active bacteria.

### ^***18***^***F-labelled arabinofuranose derivates***

**Fig. 16:**
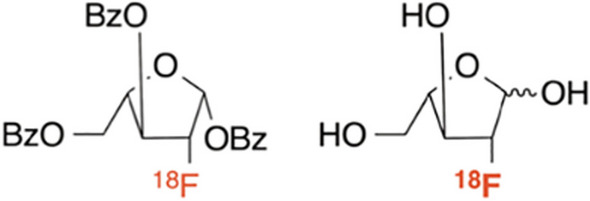
Structures of D-2-[^18^F]FAF and L-2-[^18^F]FAF (Kalita et al. 2020)

Arabinose is a monosaccharide, which plays a key role in the metabolic processes of many bacteria. Kalita et al. (2020) explored this metabolic pathway in the radiosyntheses and subsequent investigation of bacterial incorporation of four arabinofuranose-derived PET tracers, i.e. 2-deoxy-2-[^18^F]fluoro-D-arabinofuranose (D-2-[^18^F]FAF), 2-deoxy-2-[^18^F]fluoro-L-arabinofuranose (L-2-[^18^F]FAF), 5-deoxy-5-[^18^F]fluoro-D-arabinofuranose (D-5-[^18^F]FAF), and 5-deoxy-5-[^18^F]fluoro-L-arabinofuranose (L-5-[^18^F]FAF). The authors reported radiochemical yields and purities of 7.5% and 98% for D-2-[^18^F]FAF (n = 12), 10.5% and 98% for L-2-[^18^F]FAF (n = 10), 1% and 93% for D-5-[^18^F]FAF (n = 3), and 1% and 95% for L-5-[^18^F]FAF (n = 3). In vitro bacterial uptake studies of all 4 radiotracers in *E. coli* and *S. aureus* cultures were performed, which illustrated general low uptake in *S. aureus* cells and similar significantly high uptake of L-2-[^18^F]FAF and D-2-[^18^F]FAF (see Fig. 16) in *E. coli* cells. The promising results of the latter 2 tracers motivated further investigation in other bacterial pathogens. The highest bacterial uptake for L-2-[^18^F]FAF was observed in *K. pneumoniae*, *A. baumannii*, and *M. marinum*. D-2-[^18^F]FAF performed similarly to D-[methyl-^11^C]methionine with the highest bacterial uptake demonstrated in *P. mirabilis*, *S. typhimurium*, *E. faecalis* and *S. epidermidis*. It is important to note that these two tracers illustrated accumulation in both Gram-positive and Gram-negative bacteria, even though initial uptake studies in *S. aureus* were negative.

Clinical translation of these radiolabelled bacteria-unique sugars may be expected in the near future, which is highlighted by a sustained interest in these radiopharmaceuticals over the past 15 years. For example, a research report in 2022 by Kim et al. (2022) indicated that [^18^F]FDS may also have a role and function in imaging aspergillosis. Other investigations utilised [^18^F]FDS-PET as a biomarker that allows for semiquantitative visualisation of tumour-targeting bacteria. Clear-cut clinical study designs should be recommended to investigate the extended clinical role and value of these infection imaging agents.

### ^***18***^***F-labelled nitrogen mustard analogues***

**Fig. 17:**
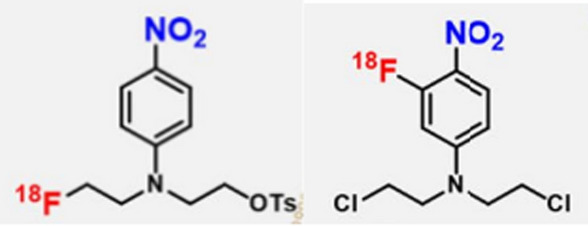
Structures of [^18^F]NTRP (left) and [^18^F]NCRP (right) (Huang et al. 2022)

Nitroreductase is an enzyme synthesised by many bacterial species and plays a role in nitrogen metabolism through the reduction of nitro groups. Huang et al. (2022) utilised the function of this bacterial enzyme for PET imaging of bacteria-specific infections through the design of two ^18^F-labelled nitrogen mustard analogues, with aromatic nitro groups. [^18^F]NTRP and [^18^F]NCRP (see Fig. 17) were produced via a fully automated radiosynthesis method.

These radiopharmaceuticals were reduced by bacterial nitroreductase and trapped within live *E. coli* and *S. aureus* cells in vitro. The authors also performed PET/CT imaging in a mouse myositis model. [^18^F]NCRP outperformed [^18^F]NTRP in terms of increased localisation in *E. coli* and *S. aureus* infected muscles, higher target-to-background ratios, uptake that correlates with the severity of the infection and distinction between sterile inflammation and infection. However, [^18^F]NCRP illustrated increased skeletal accumulation over time, which indicate defluorination of the radiopharmaceutical in vivo. Increased accumulation in the stomach and intestines over time were observed with both radiopharmaceuticals, which was most probably the result of normal microflora present in these organs.

### [^124^I]FIAU

1-(2’-deoxy-2’-fluoro-β-D-arabinofuranosyl)-5-iodouracil (FIAU) is a substrate for bacterial thymidine kinase (TK) and becomes trapped in the bacterial cell after phosphorylation. This tracer is not a newly developed radiopharmaceutical; however, a new clinical investigation was published in 2020. Previously, successful PET imaging of 8 patients with confirmed musculoskeletal infections was performed with [^124^I]FIAU, but another study reported poor specificity and image quality in patients with suspected prosthetic joint infections (Cho et al. 2020). Cho et al. (2020) aimed to clarify these ambiguous results and conducted PET/CT imaging in 14 patients with suspected musculoskeletal infections, 14 patients with suspected pulmonary infections and 3 patients with confirmed sterile inflammation due to rheumatoid arthritis (RA) as the control group. [^124^I]FIAU PET/CT images were compared to [^18^F]FDG images. Patients were scanned 1 h after [^18^F]FDG administration and 2 h after [^124^I]FIAU injection, and [^18^F]FDG PET/CT imaging was always performed first. Infection was confirmed in four of the patients with suspected musculoskeletal infection via other clinical diagnostics, but [^124^I]FIAU could only detect infection in one of these patients, resulting in a specificity of 0%, sensitivity of 25% and general accuracy of 20%, whereas the specificity, sensitivity and accuracy of [^18^F]FDG in this patient cohort were 0%, 75% and 60%, respectively. No definitive infection could be confirmed in the patients with suspected pulmonary infection via other clinical determinations, which makes it quite difficult to determine the true value of [^124^I]FIAU in these patients, but Cho et al. (2020) reported a sensitivity of 72.7% and accuracy of 72.7% ([^18^F]FDG: 0% and 0%, respectively). [^124^I]FIAU accumulation was observed in one of the 3 patients with sterile inflammatory RA. The disappointing results of this study did not warrant further clinical investigations.

### Bacterial folic acid synthesis pathway

#### *[*^*11*^*C]para-aminobenzoic acid (PABA)*

**Fig. 18:**
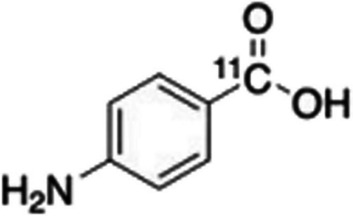
Structure of [^11^C]PABA (Ordonez et al. 2022)

*Para*-aminobenzoic acid (PABA) is utilised by many bacterial species in the synthesis of folic acid (Krátký et al. 2020). The uptake of PABA by various pathogens was investigated by Jain (2022), who reported rapid accumulation of PABA in *S. aureus* (Gram-positive), *E. coli* (Gram-negative, Enterobacterales), *P. aeruginosa* (Gram-negative, non-Enterobacterales), and mycobacteria including *Mycobacterium tuberculosis (M. tuberculosis)*. No accumulation of PABA was observed in mammalian cells or heat-killed bacteria. Ordonez et al. (2022) assessed [^11^C]*para*-aminobenzoic acid ([^11^C]PABA; see Fig. 18) PET imaging in preclinical models of myositis due to *E. coli* or *S. aureus* infection, prosthetic joint methicillin-resistant *S. aureus* (MRSA) infection, as well as vertebral discitis-osteomyelitis due to *S. aureus* infection. Specific accumulation of the radiopharmaceutical in infectious foci, with minimal uptake in sterile inflammatory foci and unaffected tissues was reported. Preclinical investigations were also performed by Parker et al. (2023) on the performance of [^11^C]PABA in vertebral discitis-osteomyelitis. They found a 20 times higher accumulation of [^11^C]PABA in infectious foci when compared to uptake in normal tissues and a high sensitivity to detect bacterial counts as low as 10^3^ cfu. Parker et al. (2023) also aimed to distinguish between *S. aureus* and *E. coli*, as *S. aureus* is the main causative pathogen of vertebral discitis-osteomyelitis, which was possible with complimentary imaging using [^11^C]PABA and [^18^F]FDS.

Ordonez et al. (2022) published first-in-human [^11^C]PABA PET/CT imaging in five healthy human volunteers to investigate its safety and biodistribution. No adverse effects were detected up to 25 days after imaging. Favourably, rapid renal excretion of the radiopharmaceutical and low background activities in other organs was observed. These promising results warrant further clinical studies in patients with infection.

### ***Ethyl 2-[***^***18***^***F]F-4-nitrobenzoate (2-[***^***18***^***F]F-ENB)***

**Fig. 19:**
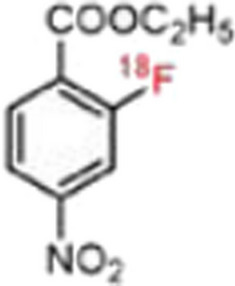
Structure of 2-[^18^F]F-ENB (Li et al. 2020)

Li et al. (2020) previously synthesised 2-[^18^F]F-PABA and investigated its accumulation in *S. aureus* infections. However, the authors found that 2-[^18^F]F-PABA was rapidly metabolised in vivo via N-acetylation, which the group addressed with the replacement of the aromatic amine with a nitro group, for reduction by bacterial nitroreductases. 2-[^18^F]F-PABA also underwent rapid renal excretion with low accumulation in infectious foci. The group therefore also converted carboxylate to the less polar ethyl ester. The authors ultimately synthesised 2-[^18^F]F-ENB (see Fig. 19) with high radiochemical purity (99%) within 90 min via a one-step manual process utilising ethyl 2,4-dinitro benzoate as the starting material. The ability of 2-[^18^F]F-ENB to specifically detect infection was assessed in an *S. aureus*-infected triceps rat model, using heat-killed *S. aureas* in the contralateral triceps to induce sterile inflammation. This study showed a 17-fold increase in 2-[^18^F]F-ENB accumulation in the infected muscle when compared to the inflammatory foci. Furthermore, in vivo stability studies of 2-[^18^F]F-ENB in a healthy rat model demonstrated the rapid hydrolysation of 2-[^18^F]F-ENB to the corresponding acid, 2-[^18^F]fluoro-4- nitrobenzoate (2-[^18^F]F-NB), which was responsible for the majority of the PET signals detected at 5, 30 and 60 min post-injection.

### Bacterial peptidoglycan synthesis pathway

D-amino acids are used in the synthesis of peptidoglycan, a component in the cell wall structure of both Gram-positive and Gram-negative bacteria (Kleynhans et al. 2023; Polvoy et al. 2022). ^11^C-labelled D-amino acids, like D-methyl-[^11^C]methionine (D-[^11^C]Met), D-[3-^11^C]alanine (D-[^11^C]ala) and D-[3-^11^C]alanyl-D-alanine (D-[^11^C]ala-D-ala) are known to accumulate in bacteria via this peptidoglycan salvage pathway.

### ***D-methyl-[***^***11***^***C]methionine***

**Fig. 20:**
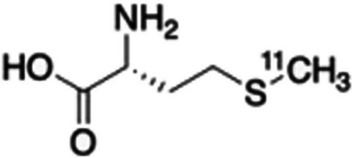
Structure of D-[^11^C]Met (Polvoy et al. 2022)

Polvoy et al. (2022) prepared D-[^11^C]Met (see Fig. 20) with the use of an automated synthesis module and performed PET/MRI dosimetry studies in 6 healthy human volunteers, as well as PET/MRI imaging in 5 patients with suspected chronic prosthetic joint infections on long-term antibiotic treatment. Dosimetry results illustrated accumulation in the liver, lung, heart and kidneys which cleared quite rapidly, but with prolonged accumulation in the liver. The authors speculated that liver accumulation of the tracer may have been due to D-amino acid metabolism in the liver. There was approximately 1.5-fold increased accumulation of D-[^11^C]Met in suspected joint infection vs contralateral joints. These results were deemed promising, but larger exploratory clinical studies in patients with confirmed infection are required to determine the role and value of D-[^11^C]Met as an infection imaging agent. Head-to-head comparative studies with current standard imaging agents, such as [^18^F]FDG or [^99m^Tc]Tc-HMPAO-labelled leukocytes, would therefore be advisable.

### ***D-[3-***^***11***^***C]alanine***

**Fig. 21:**
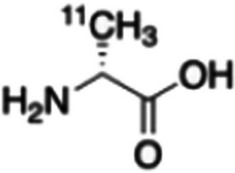
Structure of D-[^11^C]ala (Parker et al. 2020)

Parker et al. (2020) synthesised and investigated the sensitivity of D-[^11^C]ala (see Fig. 21) and D-[^11^C]ala-D-ala to detect specific bacterial species. Both radiopharmaceuticals were synthesised with a relatively low radiochemical yield (39.2 ± 3.3% and 16 ± 2.9%, respectively), but high radiochemical purity (94.6 ± 1.2% and > 95%, respectively). After evaluating their uptake in *E. coli* and *S. aureus*, D-[^11^C]ala was found to be superior and was further investigated in vitro in a wide range of bacterial species, including the main hospital-acquired pathogen—*P. aeruginosa*. The accumulation of D-[^11^C]ala in an animal model of *E. coli*- and *S. aureus*-induced myositis was evaluated to determine its ability to differentiate between active bacterial infection and sterile inflammation, with results superior to [^18^F]FDG and [^68^Ga]Ga-citrate PET. The ability of D-[^11^C]ala to detect intractable infections, such as *S. aureus*-induced vertebral-discitis osteomyelitis in a rat model and *P. aeruginosa*-induced pneumonia in a mouse model, was also studied, with promising positive results.

### ***D-5-[***^***11***^***C]glutamine***

Renick et al. (2021) synthesised d-5-[^11^C]glutamine (d-[5-^11^C]Gln) and compared this potential PET bacterial infection imaging tracer with its l-isomer, i.e. l-[5-^11^C]Gln. D-[5-^11^C]Gln demonstrated rapid renal clearance, while l-[5-^11^C]Gln resulted in much higher accumulation in other organs. The ability of d-[5-^11^C]Gln to specifically detect bacterial infections was assessed in a dual-infection mouse myositis model and illustrated significantly higher PET signal detection in *E. coli*- and MRSA-induced infections when compared to the l-isomer (*p* = 0.0004). The specific bacterial uptake of d-[5-^11^C]Gln was confirmed in another comparison study with its localisation in sterile inflammation and the accumulation of d-[5-^11^C]Gln compared with [^18^F]FDG accumulation.

### [^18^F]FMA

**Fig. 22:**
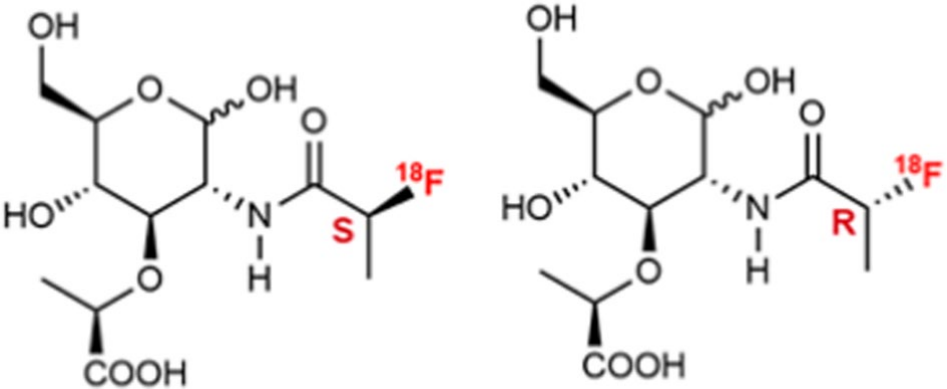
Structure of (S)-[^18^F]FMA (left) and (R)-[^18^F]FMA (right) (Lee et al. 2023)

Lee et al. (2023) mentioned a critical limitation of the ^11^C-labelled D-amino acids as the short half-life of ^11^C (20 min), which necessitates on-site cyclotron production and limits their use in the hospital setting, especially for emergency procedures. The authors therefore investigated a ^18^F-labelled tracer and focussed on N-acetyl muramic acid (NAM), which together with N-acetyl glucosamine, forms part of the carbohydrate backbone of peptidoglycan. A relatively complicated 90-min radiosynthesis involving acylation of muramic acid with 4-nitrophenyl 2-[^18^F]fluoropropionate ([^18^F]NFP) yielded the NAM-derived diastereomers, (S)-[^18^F]FMA and (R)-[^18^F]FMA (see Fig. 22) with a radiochemical yield of 35%, radiochemical purity > 99% and demonstrated stability in saline, mouse serum and human serum at 37 °C for 90 min. In vitro bacterial uptake studies of both tracers in a variety of bacterial cultures were performed with the highest uptake of (S)-[^18^F]FMA observed in Gram-negative bacteria (*E. coli*, *E. coli Xen14*, *P. aeruginosa Xen41*, *K. pneumoniae*, and *P. mirabilis*) and the highest uptake of (R)-[^18^F]FMA illustrated in Gram-positive bacteria (except *L. monocytogenes*). In vivo biodistribution studies in healthy mice revealed the kidneys and bladder as the dose-limiting organs and PET/CT imaging in mice infected with *S. aureus*, *E. coli*, and *S. epidermidis* were done with both tracers. (S)-[^18^F]FMA and (R)-[^18^F]FMA accumulated in both the bacteria-infected muscle and sterile inflammatory foci, with rapid clearance from inflamed muscle reported over time. The PET signals detected with (S)-[^18^F]FMA in *S. aureus*- and *E. coli*-infected muscle were significantly higher than the inflamed muscle. (R)-[^18^F]FMA illustrated high accumulation in *S. epidermidis*-infected muscle. Further studies are needed to investigate the differences in sensitivity of these two tracers to different pathogens and to clarify the ex vivo and in vivo bacterial uptake results.

### ^***125***^***I-labelled anti-C3d monoclonal antibody***

*M. tuberculosis* and *S. aureus* are able to generate C3d, which is a terminal fragment of the third component of complement, C3, involved in the initiation of the body’s immune response against these pathogens via antigen–antibody binding. Foss et al. (2019) utilised this aspect of the immune response against *M. tuberculosis* and *S. aureus* and radiolabelled the 3d29 monoclonal antibody with iodine-125 ([^125^I]iodo-3d29-mAb), which recognises covalently-bound C3d on the bacteria cells. A radiochemical purity of ≥ 92% was achieved. [^125^I]iodo-3d29-mAb-SPECT/CT imaging was subsequently performed in a *M. tuberculosis* infected mouse model. SPECT/CT imaging was performed at 24 and 48 h post administration of [^125^I]iodo-3d29-mAb. In healthy mice (n = 2), radioactivity was observed in the spleen and thyroid; however, infected mice (n = 2) illustrated focal uptake in the lungs, and also accumulation in the enlarged spleen, thyroid and kidneys. Thyroid accumulation of the tracer may insinuate the occurrence of unbound ^125^I-activity in vivo. These results suggest that [^125^I]-iodo-3d29-mAb-SPECT/CT imaging may be useful in the detection and localisation of active *M. tuberculosis* and to possibly assess treatment efficacy. However, much larger and well-structured preclinical studies are needed for confirmation of these early findings.

### Radiolabelled antibiotics

Radiolabelled antibiotics have gained some interest as bacteria-specific imaging agents over the past few years (Gouws et al. 2022; Koźmiński et al. 2021). Initially, [^99m^Tc]Tc-ciprofloxacin was investigated, but with poor broad spectrum SPECT infection imaging results, due to the increasing antimicrobial resistance issue and low radiochemical yield (Signore et al. 2023; Fang et al. 2020; Naqvi et al. 2019). Potential challenges with antibiotic-based radiopharmaceuticals are the imaging of antibiotic-resistant bacteria and the fact that these radiopharmaceuticals may be too specific to a narrow spectrum of bacteria, with resultant false-negative results for infection (Signore et al. 2023; Gouws et al. 2022; Lee et al. 2022). A detailed review of all relevant antibiotic-based PET radiopharmaceuticals investigated over the last few years was provided by Gouws et al. (2022), which also included those PET radiopharmaceuticals investigated for the evaluation of pharmacokinetic, pharmacodynamic and pharmacologic principles of certain drugs used for the treatment of infections. The most recent advances in radiolabelled antibiotics for bacteria-specific infection imaging reported in literature (since 2019) are summarised below.

### ^***99m***^***Tc- and ***^***68***^***Ga-labelled ciprofloxacin***

**Fig. 23:**
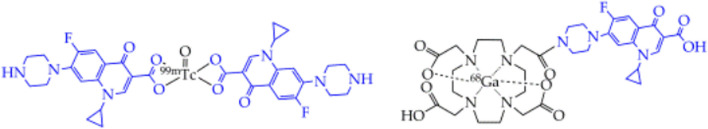
Structures of [^99m^Tc]Tc-CIP (left) and [^68^ Ga]Ga-DOTA-CIP (right) (Koźmiński et al. 2021)

Diabetic foot syndrome is a potentially severe complication of diabetes due to tissue ischaemia caused by hyperglycaemia and resultant blood clots. Tissue ischaemia causes hypoxia and necrosis, which can eventually lead to chronic infections. Ciprofloxacin (CIP) is a quinolone antibiotic that causes bacterial death due to the inhibition of DNA synthesis as a result of topoisomerase II disruption (Pham et al. 2019). Koźmiński et al. (2021) radiolabelled ciprofloxacin with ^99m^Tc directly and with ^89^Ga via DOTA-NHS as chelator (see Fig. 23). The synthesis of the precursor, DOTA-CIP (using 1 mg CIP as starting material), was quite simple but lengthy (took approximately 12 h to complete); however, the subsequent radiosynthesis seemed straightforward and fast by way of a 20-min incubation period at 95 °C to form [^68^Ga]Ga-DOTA-CIP, and 15 min incubation at 95 °C to form [^99m^Tc]Tc-CIP (using a lyophilised kit containing 2 mg CIP as starting material). The radiochemical yield and radiochemical purity of both radiopharmaceuticals were > 90%. A transchelation challenge as well as stability studies in human serum for up to 4 h were performed for both radiopharmaceuticals. The stability of [^99m^Tc]Tc-CIP in human serum was relatively low (45%), but [^68^Ga]Ga-DOTA-CIP remained almost completely intact. However, the accumulation of [^99m^Tc]Tc-CIP in *S. aureus* and *P. aeruginosa* cultures were significantly higher than [^68^Ga]Ga-DOTA-CIP, with the highest uptake observed in *S. aureus* (12.75 ± 1.02% after 60 min binding time). Clinical use included SPECT/CT imaging performed 4 h after administration of [^99m^Tc]Tc-CIP in patients with confirmed diabetic foot syndrome and suspected bacterial infection in the affected foot, yielding promising results. However, the low stability and the large amount of CIP starting material for labelling (2 mg per kit), as well as its low specificity, bacteria-binding capabilities and the issue of antimicrobial resistance reported in other studies (Signore et al. 2022), hampered its translation to routine clinical use.

### ^***99m***^***TcN-labelled ciprofloxacin xanthate (CPF2XT)***

**Fig. 24:**
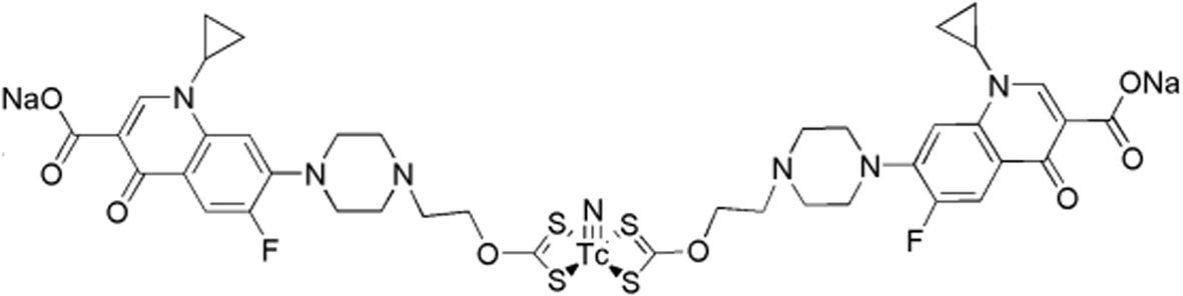
Structure of [^99m^TcN]Tc-CPF2XT (Fang et al. 2020)

The [^99m^TcN]^2+^ core forms stable conjugates with sulphur groups in ligands such as dithiocarbamates and xanthates. Fang and colleagues (Fang et al. 2020) therefore developed a ciprofloxacin xanthate (CPF2XT) and labelled it with [^99m^TcN]^2+^ for bacteria-specific SPECT/CT imaging (see Fig. 24).

Optimal labelling parameters were determined as a CPF2XT amount of 5mg, [^99m^TcN]^2+^ pH of between 8 and 9, and incubation at room temperature for 30 min, to deliver labelling yields > 90%. The stability of [^99m^TcN]Tc-CPF2XT remained high (90%) in mouse serum at 37 °C, over 6 h. Specific binding of [^99m^TcN]Tc-CPF2XT in *S. aureus* cell cultures was observed and confirmed by a significant accumulation in *S. aureus*-infected abscesses in mice (background radioactivity clearance over 30 min to 4 h after administration). Significantly lower tracer accumulation was seen in sterile abscesses in vivo. When compared to [^99m^Tc]Tc-ciprofloxacin, net accumulation of [^99m^TcN]Tc-CPF2XT in infectious foci was almost double and superior target-to-background ratios were observed, however, the overall tracer signal detected in the infection site was suboptimal to obtain high quality images. This aspect can be further investigated as the latter was probably due to target saturation (using such large amounts of starting material may cause competing cold precursor in the formulation).

### ^***99m***^***Tc-labelled norfloxacin-based tracers***

**Fig. 25:**
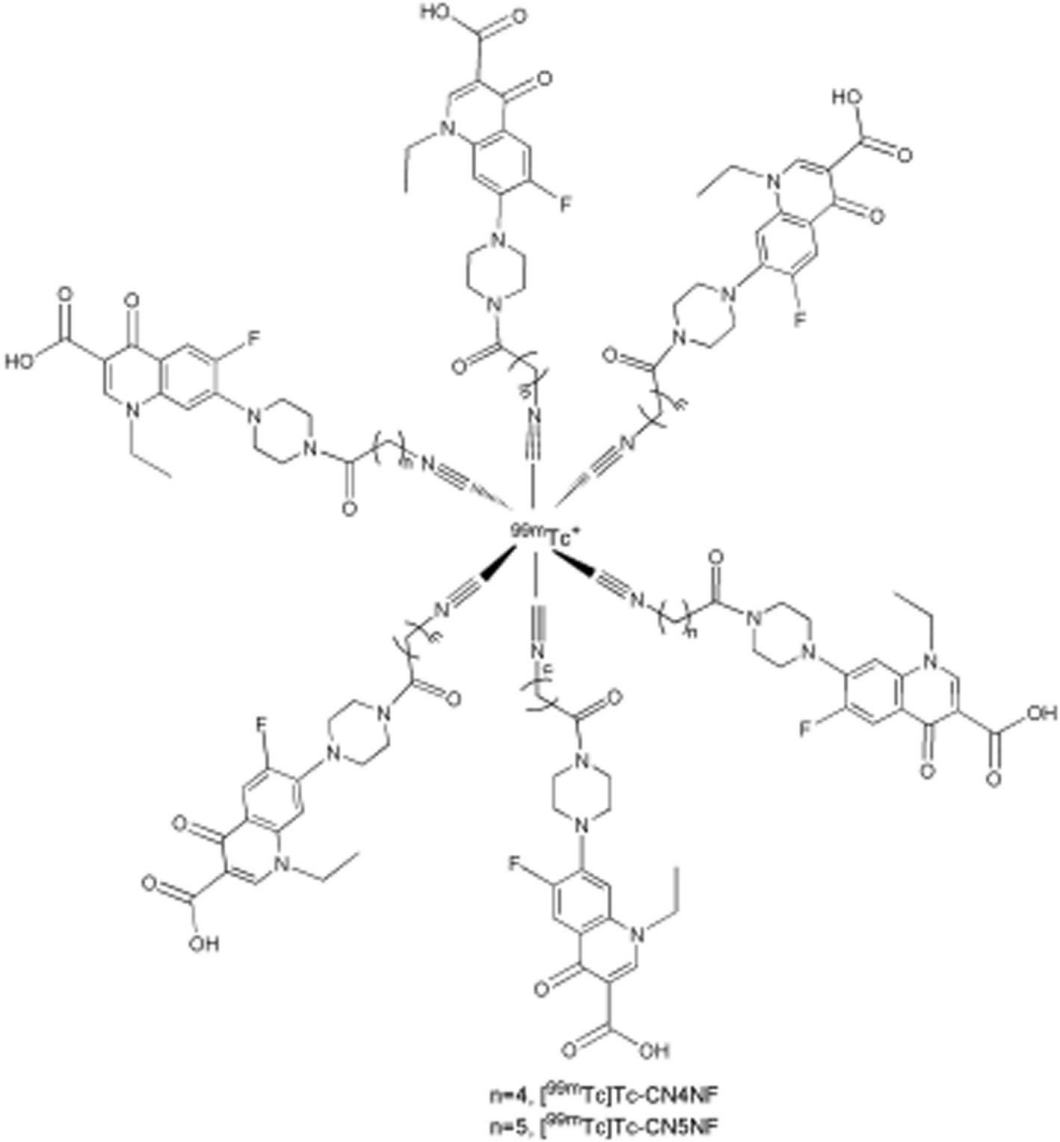
The structure of [^99m^Tc]Tc-CNnNF (n = 4 or 5) (Fang et al. 2021)

Norfloxacin is a quinolone broad-spectrum antibiotic, which has been labelled with the [^99m^Tc][Tc(V)N]^2+^ core, but this tracer demonstrated significant accumulation in the liver and lungs. Fang et al. (2021) utilised the possibility of the piperazinyl group of norfloxacin to bind to isonitrile (CN-R)-containing active esters and synthesised two norfloxacin derivatives with isonitrile groups (CN4NF and CN5NF), radiolabelled with ^99m^Tc (see Fig. 25). The rationale for this approach was based on the characteristic of isonitrile to form stable octahedral complexes with the [^99m^Tc]Tc(I) core.

The radiosynthesis of [^99m^Tc]Tc-CN4NF and [^99m^Tc]Tc-CN5NF yielded high radiochemical yields and purities (> 90%) without the need for further purification. The tracers remained stable for up to 6 h in saline and mouse serum, and demonstrated specific in vitro uptake by S. aureus, which was significantly reduced in the presence of norfloxacin. In vivo biodistribution studies in an *S. aureus*-induced myositis mouse model, with a turpentine-induced abscess to simulate sterile inflammation, illustrated accumulation of [^99m^Tc]Tc-CN4NF and [^99m^Tc]Tc-CN5NF in both infectious and inflammatory foci, but with retention of the tracer at the infection site. [^99m^Tc]Tc-CN5NF outperformed [^99m^Tc]Tc-CN4NF in displaying higher target-to-background ratios and was therefore further investigated with SPECT/CT imaging in an infection mouse model. Accumulation of [^99m^Tc]Tc-CN5NF in infectious foci was 2-fold higher than its accumulation in sterile inflammation.

Jiang et al. (2023) aimed to improve the target-to-background ratio of ^99m^Tc-labelled norfloxacin-based tracer accumulation with the synthesis of a norfloxacin 6-hydrazinoicotinamide (HYNIC) derivative (HYNICNF), and utilised different coligands for ^99m^Tc-labelling. [^99m^Tc]Tc-tricine-TPPTS-HYNICNF, [^99m^Tc]Tc-tricine-TPPMS-HYNICNF, and [^99m^Tc]Tc-EDDA-HYNICNF were produced and demonstrated good stability and specific in vitro uptake by *S. aureus* cell cultures. [^99m^Tc]Tc-EDDA-HYNICNF delivered the best in vitro results and were further investigated in an *S. aureus*-induced myositis mouse model, with similar results to Fang et al. (2021).

### ***[***^***99m***^***Tc]Tc-ertapenem***

**Fig. 26:**
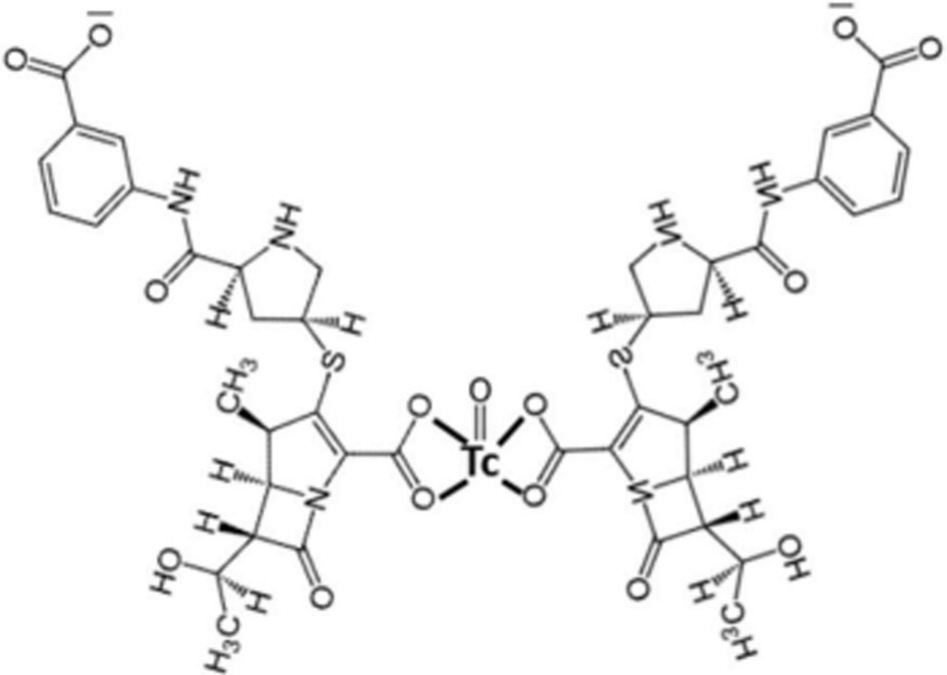
Structure of [^99m^Tc]Tc-ertapenem (Naqvi et al. 2022)

Naqvi et al. (2022) developed and performed preclinical investigations on [^99m^Tc]Tc-ertapenem (see Fig. 26) as a potential bacteria-specific SPECT infection imaging agent. Ertapenem is a carbapenem antibiotic with efficacy against multi-drug resistant bacteria causing deep-seated infections in the abdominal space and urinary tract. Ertapenem binds to penicillin-binding proteins, which are expressed by many bacterial species, but to a greater extent by *E. coli*. The optimised labelling parameters for [^99m^Tc]Tc-ertapenem featured a ligand concentration of 0.5 mg/mL, reducing agent (stannous chloride) concentration of 10 µg/mL, ^99m^Tc-activity of 2 mCi, a reaction pH of 10 and incubation for 10 min at room temperature. The radiopharmaceutical showed good stability in human serum (89% after 3 h and 82% after 6 h incubation in vitro). The authors reported significantly higher accumulation of [^99m^Tc]Tc-ertapenem in *S. aureus*- and *E.* coli-infected muscle tissue in rats, when compared to inflammatory tissue foci. Furthermore, [^99m^Tc]Tc-ertapenem accumulation in the *S. aureus*- and *E. coli*-infected thigh muscles of a rabbit model was noted as early as 30 min after radiopharmaceutical administration, with slightly elevated uptake in inflamed thigh muscles, which the authors attributed to secondary infection processes. This pilot study warrants further preclinical and clinical investigations.

### ^***99m***^***Tc- and ***^***18***^***F-labelled linezolid***

Linezolid is an oxazolidinone antibiotic indicated for a variety of difficult-to-treat Gram-positive bacterial infections, including vancomycin-resistant enterococcol infections, drug-resistant TB and MRSA. Linezolid is able to cross the blood–brain barrier, which El-Kawy et al. (2023) utilised to develop a tracer for specific bacterial brain abscess imaging, i.e. [^99m^Tc]Tc-linezolid, via the straightforward stannous chloride direct complexation radiolabelling method. A high yield of 97% was achieved. [^99m^Tc]Tc-linezolid performed well in in vitro bacterial uptake assays and demonstrated specific in vivo accumulation in an MRSA brain infection animal model.

**Fig. 27:**
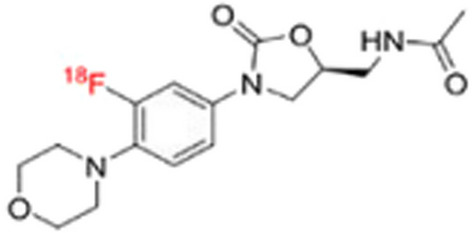
Structure of ^18^F-labelled linezolid (Mota et al. 2020b)

Mota et al. (2020b) synthesised [^18^F]linezolid (see Fig. 27) via the substitution of the existing fluorine atom in linezolid with ^18^F, utilising a copper-mediated method for the radiofluorination of boronates.

In vitro bacterial uptake studies with [^18^F]linezolid in *M. tuberculosis* cell cultures did not demonstrate any significant uptake and in vivo tracer accumulation studies in a pulmonary *M. tuberculosis*-infected mouse model demonstrated rapid tracer distribution to all major organs and high, similar accumulation in both *M. tuberculosis*-infected and healthy lungs. The authors reported that although [^18^F]linezolid did not seem to be a very promising TB PET imaging agent at that stage, further investigations are required, and the tracer may potentially aid in linezolid concentration measurement of various tissues, which may also play a role in the development of future oxazolidinone antibiotics.

### ***[***^***18***^***F]pretomanid***

**Fig. 28:**
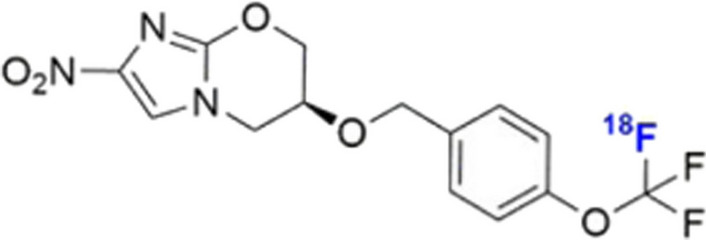
Structure of [^18^F]pretomanid (Mota et al. 2022)

Pretomanid is an antitubercular nitroimidazole antibiotic, which forms part of the treatment regimen for multi-drug resistant TB (MDR). As antibiotic dosing recommendations are based on the plasma concentration of the drug and the drug concentration in infection foci are not generally known, Mota et al. (2022) developed [^18^F]pretomanid (see Fig. 28) with the aim of noninvasively assessing distribution of the drug to tissues in TB meningitis mouse and rabbit models via quantification on PET/CT images. [^18^F]pretomanid was produced via halogen exchange ^18^F-fluorination of a synthesised aryl-bromodifluoromethoxyl precursor with a radiochemical yield of approximately 5% and a radiochemical purity of > 98%. The authors also optimised the radiosynthesis method to an automated cGMP synthesis of [^18^F]pretomanid for use in humans with a radiochemical yield of approximately 6% and a radiochemical purity ≥ 95%. In vitro stability studies revealed high stability of [^18^F]pretomanid in mouse, rabbit and human serum over 3 h with no defluorination observed. In vivo biodistribution studies in a pulmonary TB mouse model demonstrated rapid distribution of [^18^F]pretomanid to all major organs, no increase in bone uptake over time, evidence of renal and hepatobiliary excretion, low tracer accumulation in muscle and high accumulation in brown adipose tissue which washed out over time. Interestingly, [^18^F]pretomanid accumulation was lower in infected lung tissue than in healthy lung tissue. These in vivo biodistribution results were confirmed in a rabbit model. Accumulation of [^18^F]pretomanid in TB meningitis were investigated in mouse and rabbit models, which demonstrated excellent brain penetration of the tracer but lower accumulation in the brain lesions. [^18^F]pretomanid-PET imaging was also performed in 6 healthy human volunteers, which yielded similar biodistribution results as were obtained in the animal studies and did not results in any adverse or pharmacological effects. The authors concluded that [^18^F]pretomanid-PET is a promising agent for the noninvasive pretomanid concentration measurement of various tissues.

### ^***18***^***F-vancomycin-based tracers***

**Fig. 29:**
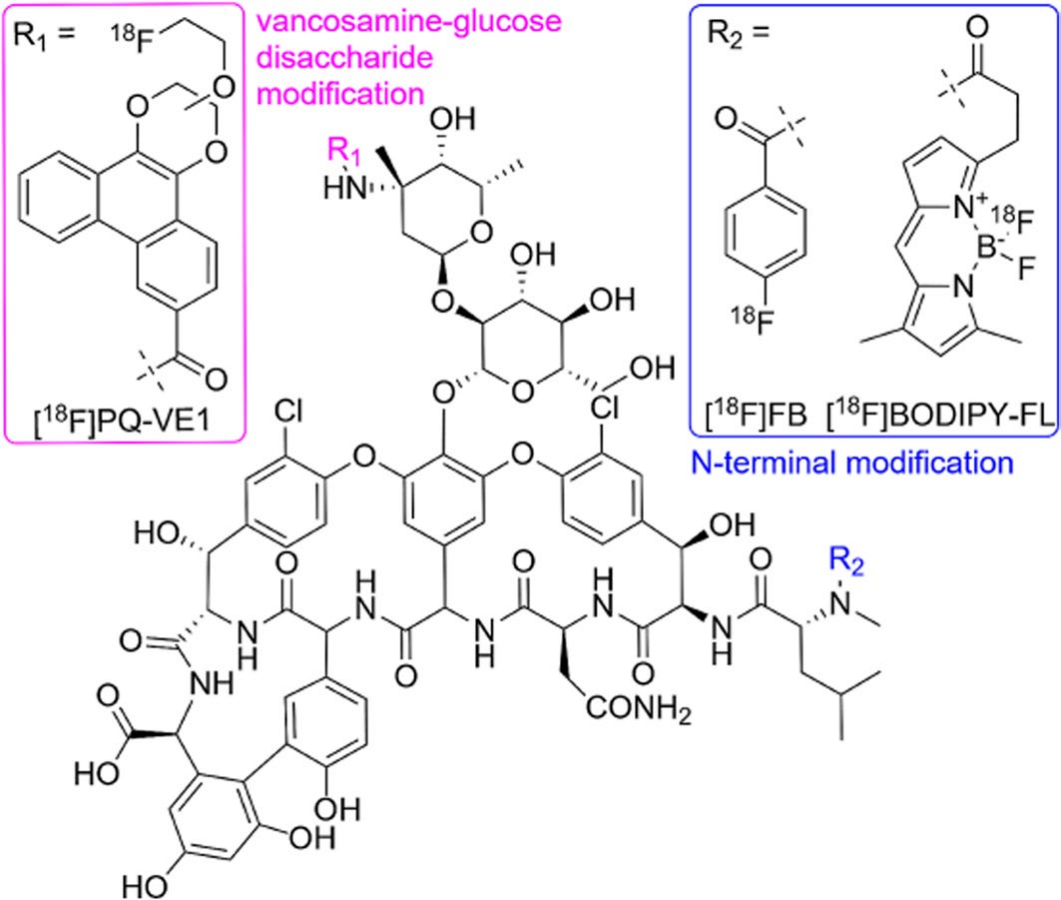
Structures of vancomycin, [^18^F]FB-vancomycin, [^18^F]BODIPY-FL-vancomycin and [^18^F]PQ-VE1-vancomycin (Spoelstra et al. 2024)

Vancomycin is an antibiotic that inhibits bacterial cell-wall synthesis by blocking glycopeptide polymerisation via its binding to D-alanyl-D-alanine. The major pharmacological action of vancomycin is against Gram-positive bacteria. Spoelstra et al. (2024) utilised these characteristics to synthesise and investigate three vancomycin-based tracers labelled with ^18^F, i.e. [^18^F]FB-vancomycin, [^18^F]BODIPY-FL-vancomycin and [^18^F]PQ-VE1-vancomycin (see Fig. 29), for potential Gram-positive bacteria-specific PET infection imaging. There are two nucleophilic amine groups on vancomycin that allow binding with electrophilic ^18^F—a primary amine on the vancosamine-glucose disaccharide to which [^18^F]PQ-VE1 binds, and a secondary amine in the peptide backbone which [^18^F]FB and [^18^F]BODIPY-FL interacts with.

All 3 tracers were synthesised with radiochemical purities > 95%. The time for radiosynthesis differed slightly for each tracer, ranging from approximately 83 to 109 min, and the highest radiochemical yield of about 12% was obtained for [^18^F]FB-vancomycin (± 1% for [^18^F]BODIPY-FL-vancomycin and 3% for [^18^F]PQ-VE1-vancomycin). Both [^18^F]BODIPY-FL-vancomycin and [^18^F]PQ-VE1-vancomycin illustrated > 90% stability in PBS and human serum over 120 min, whereas [^18^F]FB-vancomycin was stable in PBS but not in human serum. The instability of [^18^F]FB-vancomycin also meant that it did not illustrate any in vitro bacterial uptake. In vitro bacterial uptake studies for [^18^F]BODIPY-FL-vancomycin and [^18^F]PQ-VE1-vancomycin demonstrated significantly high uptake in *S. aureus* cell cultures (which was relatively resistant to competitive inhibition with vancomycin), as well as other Gram-positive bacteria. In vivo biodistribution studies and PET imaging in a healthy mouse model illustrated the highest accumulation of [^18^F]BODIPY-FL-vancomycin in the kidneys and bladder, with some tracer accumulation also reported in the liver, bone and lungs. [^18^F]PQ-VE1-vancomycin demonstrated the highest accumulation in the spleen and high signals were also detected in the blood pool, with some accumulation in the kidneys and bladder.

### ***Radiolabelled antifolates—***^***11***^***C- and ***^***18***^***F-labelled trimethoprim***

**Fig. 30:**
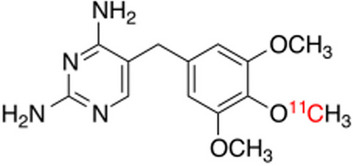
Structure of [^11^C]TMP (Lee et al. 2022)

Trimethoprim (TMP) is a broad-spectrum antibiotic that binds to and inhibits dihydrofolate reductase which prevents bacterial folate activation and therefore bacterial growth of Gram-positive, Gram-negative and mycobacterial species (Gouws et al. 2022; Welling et al. 2019). A first-in-human study on the biodistribution of [^11^C]TMP was reported by Doot et al. (2019). The latest advances were reported by Lee et al. (2022), highlighting the reported promising clinical results of [^11^C]TMP (see Fig. 30) in both TMP-sensitive and TMP-resistant bacteria in an exploratory patient case series (n = 4). The patients that were investigated included a patient with metastatic lung adenocarcinoma which did not show significant [^11^C]TMP uptake, illustrating the specificity of [^11^C]TMP for infection. Two patients with cystic fibrosis were investigated and corresponding [^11^C]TMP uptake with foci of pneumonia was reported in the one patient, which still localised the radiopharmaceutical after antibiotic treatment, indicating its sensitivity. Increased [^11^C]TMP uptake was also seen in the affected lung of the other patient, but a follow-up scan could not be performed. The last patient investigated had discitis osteomyelitis which also illustrated increased [^11^C]TMP localisation in the affected vertebrae. In addition to the latter pilot study, Phase 1 clinical trials concerning the performance and safety of [^11^C]TMP and [^18^F]fluoropropyl-trimethoprim as bacteria-specific PET imaging agents are currently underway (Akter et al. 2023; Kleynhans et al. 2023).

A summary of the selected radiopharmaceuticals investigated for bacterial infection imaging over the last 5 years is provided in Table 1.

**Table 1:** A summary of the selected (most recent) bacterial infection imaging agents under investigation

	Radiopharmaceutical	Imaging technique	Radioisotope production	Radiosynthesis approach	Reported accumulation in sterile inflammatory foci	Specific bacterial uptake	References
Bacterial siderophore targeting	[^68^Ga]Ga-DFO-B	PET	^68^Ge/^68^Ga generator	Simple manual synthesis	None	Broad-spectrum bacterial uptake. High accumulation in *P. aeruginosa* and *S. aureus* reported	Petrik et al. (2021)
	^68^Ga-labelled cyclen-based artificial siderophore ([^68^Ga]Ga-compound-7)	PET	^68^Ge/^68^Ga generator	Total chemical synthesis, followed by automated radiolabelling	Low uptake in sterile inflammation, with rapid washout	Broad-spectrum bacterial uptake. High accumulation in *E. coli* reported	Peukert et al. (2021)
	[^68^Ga]Ga-ORNB-C6	PET	^68^Ge/^68^Ga generator	Simple manual synthesis	None	High accumulation in *B. multivorans* and significantly lower uptake in *S. aureus* and *P. aeruginosa*. No accumulation in *E. coli*	Bendova et al. (2023)
	[^68^Ga]Ga-RMA693 (enterobactin-based siderophore)	PET	^68^Ge/^68^Ga generator	Total chemical synthesis, followed by manual radiolabelling	None	Specific uptake in *E. coli* ATCC25922 strain	Margeta et al. (2024)
	[^68^Ga]Ga-ferrirubin	PET	^68^Ge/^68^Ga generator	Simple manual synthesis	None	High uptake in *S. aureus, P. aeruginosa* and *K. pneumoniae*, with negligible uptake in *E. coli*	Krasulova et al. (2024)
	[^64^Cu]Cu-YbT	PET	Cyclotron—^64^Ni(p,n)^64^Cu	Manual labelling	None	Ferric Ybt uptake A receptor-expressing bacteria—*E. coli* UTI89, *E. coli* Nissle WT and *Klebsiella pneumoniae*	Siddiqui et al. (2021)
Antimicrobial peptides	[^68^Ga]Ga-NOTA/DOTA-UBI29-41	PET	^68^Ge/^68^Ga generator	Automated synthesis and in-house production reported	Low uptake in sterile inflammation, with rapid washout	Broad-spectrum bacterial uptake (including *P. aeruginosa*, *S. aureus* and *Streptococcus pyogenes)*	Santos et al. (2024), Boddeti and Kumar (2021), Marjanovic-Painter et al. (2023), Vilche et al. (2019), Roux et al. (2020)
	[^68^Ga]Ga-DOTA-GF-17	PET	^68^Ge/^68^Ga generator	Manual labelling	High initial uptake in sterile inflammation, with washout over time	Broad-spectrum bacterial uptake. Optimal uptake in *S. aureus* reported at 45 min and *P. aeruginosa* at 120 min	Chopra et al. (2019)
	[^99m^Tc]Tc-HBD-3	SPECT	^99^Mo/^99m^Tc generator	Manual labelling	Low	Broad-spectrum bacterial uptake, but accumulation in *S. aureus* > *E. coli*	Follacchio et al. (2019)
	[^99m^Tc]Tc-HYNIC/EDDA-MccJ25	SPECT	^99^Mo/^99m^Tc generator	Manual labelling	Moderate	Demonstrated for *E. coli*	Tehrani et al. (2021)
	[^99m^Tc]Tc-HYNIC-polymyxin B	SPECT	^99^Mo/^99m^Tc generator	Manual synthesis	Moderate	High uptake in Gram-negative bacteria	Auletta et al. (2021)
	[^99m^Tc]Tc-UBI29-41-2-APBA	SPECT	^99^Mo/^99m^Tc generator	Manual synthesis	Low uptake in sterile inflammation, with rapid washout	Demonstrated for *S. aureus*	Mitra et al. (2022)
	[^99m^Tc]Tc-CN5UBI29-41 (isocyanide derivative)	SPECT	^99^Mo/^99m^Tc generator	Manual synthesis	Significantly higher uptake in infection than in inflammation	Demonstrated for *S. aureus*	Jiang et al. (2024)
	[^64^Cu]Cu-DOTA-HLys-AB1	PET	Cyclotron—^64^Ni(p,n)^64^Cu	Manual labelling	Not reported	Gram-positive bacteria	Aweda et al. (2019)
Bacteria-unique sugars	[^18^F]FDS	PET	Cyclotron—^18^O(p,n)^18^F	Kit-based preparation from [^18^F]FDG	Low	Specific for Enterobacterales infections – drug-susceptible as well as MDR strains	Ordonez et al. (2021), Mota et al. (2021)
	[^18^F]FDM and [^18^F]FSK	PET	Cyclotron—^18^O(p,n)^18^F	Simple manual synthesis from [^18^F]FDG	Low to moderate	High uptake in *S. aureus, A baumannii* and *K. pneumoniae*, and low uptake in *E. coli*	Sorlin et al. (2023)
	[^18^F]FMH	PET	Cyclotron—^18^O(p,n)^18^F	Simple manual synthesis	Low	Broad-spectrum uptake demonstrated, including MRSA	Takemiya et al. (2019)
	[^18^F]fluoromaltotriose	PET	Cyclotron—^18^O(p,n)^18^F	Manual synthesis	Low	Broad-spectrum. Uptake demonstrated for *E. coli*	Gabr et al. (2020)
	L-2-[^18^F]FAF and D-2-[^18^F]FAF (arabinofuranose derivatives)	PET	Cyclotron—^18^O(p,n)^18^F	Manual synthesis	Not reported	Broad-spectrum	Kalita et al. (2020)
Targeting bacterial nitro-reductase	^18^F-labelled nitrogen mustard analogues ([^18^F]NCRP and [^18^F]NTRP)	PET	Cyclotron—^18^O(p,n)^18^F	Automated synthesis	Low to Moderate	Broad-spectrum. Uptake in *E. coli* > *S. aureus*	Huang et al. (2022)
Targeting bacterial folic acid synthesis	[^11^C]PABA	PET	Cyclotron—^14^N(p,α)^11^C	Manual synthesis described by Holt et al. (2019)	Almost negligible	Broad-spectrum. Uptake investigated in *E. coli*, *S. aureus* and MRSA	Ordonez et al. (2022), Parker et al. (2023), Holt et al. (2019)
	[^18^F]F-ENB	PET	Cyclotron—^18^O(p,n)^18^F	Manual synthesis	Low	Demonstrated for *S. aureus*	Li et al. (2020)
Targeting bacterial peptidoglycan synthesis	D-[^11^C]Met	PET	Cyclotron—^14^N(p,α)^11^C	Automated synthesis	Relatively high	Broad-spectrum	Polvoy et al. (2022)
	D-[^11^C]Ala	PET	Cyclotron—^14^N(p,α)^11^C	Manual synthesis	Almost negligible	Broad-spectrum. Uptake investigated in *E. coli*, *S. aureus* and *P. aeruginosa*	Parker et al. (2020)
	D-5-[^11^C]glutamine	PET	Cyclotron—^14^N(p,α)^11^C	Manual synthesis	Low	High uptake demonstrated in *E. coli* and MRSA	Renick et al. (2021)
	[^18^F]FMA	PET	Cyclotron—^18^O(p,n)^18^F	Manual synthesis	High with rapid washout	Broad spectrum uptake. Highest uptake of (R)-[^18^F]FMA in Gram-positive bacteria and highest uptake of (S)-[^18^F]FMA in Gram-negative bacteria	Lee et al. (2023)
Immune response specific to *M. tuberculosis*	[^125^I]iodo-3d29-mAb	SPECT	Nuclear reactor—^24^Xe(n,γ)^125^Xe → ^125^I via electron capture	Manual labelling via the Iodogen™ method	Not reported	*M. tuberculosis*	Foss et al. (2019)
Radiolabelled antibiotics	[^99m^Tc]Tc-N-CPF2XT	SPECT	^99^Mo/^99m^Tc generator	Manual labelling	Moderate	Broad-spectrum. Uptake demonstrated in *S. aureus*	Fang et al. (2020)
	^99m^Tc-labelled norfloxacin-based tracers	SPECT	^99^Mo/^99m^Tc generator	Manual synthesis	High	Demonstrated for *S. aureus*	Fang et al. (2021; Jiang et al. (2023)
	[^99m^Tc]Tc-ertapenem	SPECT	^99^Mo/^99m^Tc generator	Manual labelling	Low	Broad-spectrum. Uptake investigated in *E. coli* and* S. aureus*	Naqvi et al. (2022)
	[^99m^Tc]Tc-linezolid	SPECT	^99^Mo/^99m^Tc generator	Simple manual synthesis	Not reported	Demonstrated in MRSA brain infection	El-Kawy et al. (2023)
	[^18^F]linezolid	PET	Cyclotron—^18^O(p,n)^18^F	Manual synthesis	High	No specific bacterial uptake	Mota et al. (2020b)
	[^18^F]pretomanid	PET	Cyclotron—^18^O(p,n)^18^F	Manual synthesis as well as the development of an automated cGMP synthesis method	High	No specific bacterial uptake	Mota et al. (2022)
	^18^F-vancomycin-based tracers	PET	Cyclotron—^18^O(p,n)^18^F	Manual synthesis	Moderate to high	Significantly high uptake demonstrated in S. aureus and other Gram-positive bacteria	Spoelstra et al. (2024)
	[^11^C]TMP	PET	Cyclotron—^14^N(p,α)^11^C	Manual labelling	Low	Broad-spectrum. Uptake reported in both TMP-susceptible and TMP-resistant bacteria	Giraudo et al. (2020), Welling et al. (2019)

## Conclusion

With the recent focus-shift to personalised medicine and the continuously growing global threat of infection and antimicrobial resistance, researchers have been utilising our improved understanding of pathology and bacteria in their attempts to develop more infection-specific Nuclear Medicine imaging agents for earlier and accurate diagnosis, localisation and evaluation of antibiotic treatment efficacy, than the infection imaging agents currently available. It is evident that a great deal of effort has gone into the development of new radiopharmaceuticals for bacterial infection imaging over the last few years, with remarkable progress in preclinical investigations. However, translation to clinical trials, and eventually clinical Nuclear Medicine practice, is apparently slow.

Similar to other areas of Nuclear Medicine, crucial challenges that may delay the clinical application of emerging radiopharmaceuticals for infection imaging include the high cost of clinical trials, followed by the complexity of compound registration for pharmaceutical use. More specifically, the immunogenic potential of antibody fragments and peptides, and the dose limitations for narrow-spectrum bacteria-specific radiolabelled antibiotics further narrow the area of application.

The debate on whether a tracer selective for Gram-negative as opposed to Gram-positive bacterial infections (or vice versa), or a tracer able to detect both Gram-negative and Gram-positive bacterial infections in a sensitive manner is the ultimate goal is not resolved. The preferred tracer would be dependent on the clinical scenario, where a tracer able to detect all or most pathogenic microorganisms will be ideal to distinguish infection from other processes. On the other hand, tracers selective for important categories of pathogenic microorganisms (e.g. [^18^F]FDS for Enterobacteriaceae) will play a major role to non-invasively identify specific bacterial strains for the selection of the most appropriate, effective antimicrobial therapy (Auletta et al. 2021; Kalita et al. 2020; Lee et al. 2023).

However, in this review, selected radiopharmaceuticals were highlighted with regard to a) their promising first-in-human application, b) significant results from well-structured conceptional clinical investigations in small patient populations, and c) evidence from early clinical trials. It can be concluded that the increasing clinical imaging using [^68^Ga]Ga-DFO-B, [^68^Ga]Ga-NOTA-UBI29-41, [^18^F]FDS, [^11^C]PABA, D-[^11^C]Met, [^11^C]TMP and [^18^F]fluoropropyl-trimethoprim will improve the noninvasive diagnostics of infection with more defined roles, in particular for [^18^F]FDS and [^18^F]fluoropropyl-trimethoprim, as they best meet the clinical requirements.

Although the specificity of radiolabelled leukocytes for infection imaging is not ideal, these radiopharmaceuticals are still considered as the gold standard for the detection and localisation of many infection-associated diseases/disease processes. It is the authors’ opinion that a more structured and harmonised preclinical setting and well-designed clinical investigations are the key to reliably evaluate the true potential of the newly proposed infection imaging agents, as already mentioned by several scientists in this field (Signore et al. 2023).

## Data Availability

Not applicable.
